# Pathomechanistic Networks of Motor System Injury in Amyotrophic Lateral Sclerosis

**DOI:** 10.2174/1570159X21666230824091601

**Published:** 2023-08-24

**Authors:** Bedaballi Dey, Arvind Kumar, Anant Bahadur Patel

**Affiliations:** 1 CSIR-Centre for Cellular and Molecular Biology (CSIR-CCMB), Hyderabad 500007, Telangana, India;; 2 AcSIR-Academy of Scientific and Innovative Research, Ghaziabad 201002, Uttar Pradesh, India

**Keywords:** Amyotrophic lateral sclerosis (ALS), motor neuron disease, pathophysiology, gene environment interaction, heterogeneity, metabolism, epigenetics, RNA modification

## Abstract

Amyotrophic Lateral Sclerosis (ALS) is the most common, adult-onset, progressive motor neurodegenerative disorder that results in death within 3 years of the clinical diagnosis. Due to the clinicopathological heterogeneity, any reliable biomarkers for diagnosis or prognosis of ALS have not been identified till date. Moreover, the only three clinically approved treatments are not uniformly effective in slowing the disease progression. Over the last 15 years, there has been a rapid advancement in research on the complex pathomechanistic landscape of ALS that has opened up new avenues for successful clinical translation of targeted therapeutics. Multiple studies suggest that the age-dependent interaction of risk-associated genes with environmental factors and endogenous modifiers is critical to the multi-step process of ALS pathogenesis. In this review, we provide an updated discussion on the dysregulated cross-talk between intracellular homeostasis processes, the unique molecular networks across selectively vulnerable cell types, and the multisystemic nature of ALS pathomechanisms. Importantly, this work highlights the alteration in epigenetic and epitranscriptomic landscape due to gene-environment interactions, which have been largely overlooked in the context of ALS pathology. Finally, we suggest that precision medicine research in ALS will be largely benefitted from the stratification of patient groups based on the clinical phenotype, onset and progression, genome, exposome, and metabolic identities.

## 1 INTRODUCTION

Amyotrophic Lateral Sclerosis (ALS) is the third most common type of neurodegenerative disorder and the most common type of ‘Motor Neurone Disease’ (MND) [1]. The global incidence of ALS currently ranges between 0.6 and 3.8 per 100,000 person-years, which is increasing over time [2]. The prevalence of ALS is approximately 4.1-8.4 per 100,00 persons, with a lifetime risk of 1 in 350 and a median onset age of 51-66 years [3]. ALS initiates focally and asymmetrically in the upper and/or lower limbs or in the bulbar regions, followed by a gradual progression to the contiguous voluntary muscles [4]. Moreover, about 28% of sporadic and 48% of familial cases of ALS display cognitive deficits associated with frontotemporal dementia (FTD) [5]. Clinical hallmarks of ‘classical’ ALS cases involve progressive and concomitant degeneration of upper (UMN) and lower motor neurons (LMN) in the motor cortex, brain stem, and spinal cord [4]. Early clinical signs of UMN and/or LMN defects are followed by a progressive paralysis that causes death by respiratory failure in ≥ 50% of patients within 2-5 years of diagnosis [6]. Major sub-clinical signatures in ALS involve atrophy of the primary motor cortex and ventral projections of the spinal cord, degeneration of the corticospinal and corticobulbar tracts, degeneration of the hypoglossal-trigeminal-phrenic nerves of the brain stem, and wasting of the somatic and bulbar skeletal muscles [7]. Underlying microstructural abnormalities include massive death of spinal α-motor neurons and varying loss of corticomotor Betz cells, with diffuse gliosis in the subcortical white matter and the grey matter of the motor cortex and spinal cord [7].

Gender-based risk of ALS incidence is approximately double for the male sex [8], with a higher mean age at diagnosis and a lower peak age of prevalence among women [9]. Interestingly, a lower survival time with a higher risk of bulbar onset has been reported in women [10]. However, these gender ratios might change with the age of incidence, both geographically and temporally [11]. A higher incidence in Europe compared to South and East Asia has been correlated with the lower prevalence of known ALS genes in Asian populations [3, 11]. The presence of a prodromal delay, respiratory or genitourinary comorbidities, gradual weight loss or poor nutritional status, and cognitive impairment or depressive disorders tend to negatively affect survival in ALS patients [3]. Based upon their representativeness across various population-based studies, the above factors can act as the demographic variants that skew the association of disease with prognostic factors.

Due to a wide variety of ‘ALS-mimic’ disorders, the use of a range of diagnostic methods involving clinical, electrophysiological, neuroimaging, and neuropathological tests is required to ascertain the ALS ‘syndrome’ [12]. A definitive diagnosis of ALS requires the revised El Escorial criteria with the revised Airlie House criteria from the World Federation of Neurology (WFN), the Awaji-Shima criteria, and the Gold Coast criteria [13-15].

As revealed by various large-scale epidemiological studies, a wide range of susceptibility factors and resistance mechanisms contribute to the variability of disease progression and survivability in ALS [4]. The complex etiology of the ALS spectrum is believed to be the result of a time-variable cross-talk between environmental risk factors and endogenous metabolic modifiers, with diverse types of genetic polymorphisms having different cytotoxic or cytoprotective effects depending on neuronal types [16].

In this review, we highlight the complex interplay of impaired cellular processes that define the pathomechanistic signature during ALS onset and progression. This majorly involves synaptic failure, neuroinflammation, dysregulated proteostasis and RNA metabolism, impaired endosomal trafficking and axonal transport, oxidative stress, DNA damage, and metabolic dyshomeostasis [17]. The complex genetic architecture of ALS dictates the molecular heterogeneity of affected cellular networks and generates a ‘pathological continuum’ of clinical phenotypes. Moreover, this review draws a focus on the multisystem nature of pathological processes, the selective vulnerability of motor neuron and myofibre subtypes, and the dysregulated neuroglial and neuromuscular networks in ALS ‘syndrome’ [18].

Additionally, hurdles in unraveling the ‘missing heritability’ of ALS pathogenesis can be overcome by looking in the direction of DNA or RNA modifications associated with the disease. Here, we describe the critical role of gene-environment interaction in disease precipitation in conjunction with the dysregulated epigenetic machinery and epitranscriptomic landscape in ALS. Finally, we suggest that the need of the hour is to discern how the pathological cellular networks work simultaneously, sequentially, or synergistically in ALS.

## 2 GENETIC RISK FACTORS OF ALS

Traditionally, over 90-95% of cases of ALS are determined to be sporadic (sALS), and only 5-10% of cases have a familial origin (fALS), usually with an autosomal dominant inheritance pattern [19]. Over the years, >50 potentially causative or disease-modifying genes associated with ALS have been identified [20]. The genetic component in 60-70% of fALS cases is known, while the heritability of sALS cases lies at 50% [21]. Approximately 50% of fALS cases and 5% of sALS cases are attributed to high-risk genes such as chromosome 9 open reading frame 72 (C9ORF72), superoxide dismutase (SOD1), TAR-DNA binding protein (TARDBP) and fused in sarcoma (FUS) [20].

A multitude of genes associated with ALS reveals shared contribution to endocytosis and vesicle transport such as alsin 2 (ALS2), vesicle membrane protein-associated protein B (VAPB), optineurin (OPTN), and valosin-containing protein (VCP), along with axonal transport and organelle traffickings such as spatacsin (SPG11) and OPTN [20, 22]. Other genes are involved in autophagy such as TANK-binding kinase 1 (TBK1), charged multivesicular body protein 2B (CHMP2B), Sequestosome 1 (SQSTM1) and OPTN, or protein degradation and unfolded protein response such as ubiquilin 2 (UBQLN2) and sigma non-opioid intracellular receptor 1 (SIGMAR1), or oxidative stress response such as SOD1, or DNA damage and RNA metabolism such as angiogenin (ANG), senataxin (SETX), heterogenous nuclear ribonucleoprotein A1/A2 (HNRNPA1/A2), C9ORF72, TARDBP, and FUS [7, 20].

Many of the fALS-associated mutations have now been reported in sALS cases, being previously undetected due to incomplete genotypic penetrance, delayed disease onset, and/or oligogenic contribution [23]. Analyses of various large-scale genome-wide association studies contribute to the ‘multiple rare variants, common disease’ hypothesis of sporadic ALS [24]. Moreover, the genetic contribution to the risk of ALS onset and the rate of progression seems to be independent of each other [25]. It is yet unclear how the mutations in individual ALS-associated genes having divergent cellular functions ultimately converge on the signature disease phenotype. It has become apparent that systematic genetic profiling of both familial and sporadic ALS patients followed by a genetic sub-classification is crucial for the development of targeted therapeutics and relevant preclinical models for drug screening.

## 3 PATHOPHYSIOLOGY OF ALS

Contiguity of the degenerative spreading through the central nervous system (CNS) is indicated by the decline in the integrity of multiple interconnected micro-circuits within and associated with the motor network [26]. These circuits involve ‘determinants’ such as the quadripartite network of neuron-oligodendrocyte-astrocyte-microglia cell types, the neuromuscular synapse, the inhibitory feedback connections with interneurons, as well as ‘contributors’ across the neurovascular units such as lymphocytes and pericytes [26]. Here, we discuss the currently available knowledge of the pathophysiological mechanisms of ALS.

### 3.1 Multifaceted Pathology of Synaptic Failure

ALS is often regarded as a synaptopathy, wherein motor degeneration is preceded by a loss of synaptic scaling and plasticity [26, 27]. This occurs with concomitant regression and degeneration of synaptic architecture and apical dendrites [27, 28].

#### 3.1.1 Effects on Motor Neurons

Accumulation of mutant protein aggregates in astroglia (*e.g.*, SOD1, FUS) has been found to hyperactivate nuclear factor kappa B (NF-κB) and tumor necrosis factor α (TNF-α) signaling, which disrupts synaptic scaling [29]. It works through the astrocyte-mediated upregulation of the Ca^2+^ permeable types of the α-amino-3-hydroxy-5-methyl-4-isoxazole propionic acid (AMPA) glutamate receptors like GluR1, with a concomitant increase in the intrinsically Ca^2+^ -permeable N-methyl-D-aspartic acid (NMDA) glutamate receptor subunits like GluN3B in postsynaptic neurons [30]. Upregulation of metabotropic glutamate receptors like mGlu1 or mGlu5 in presynaptic neurons or astroglia, respectively; along with activation of astroglial serotonergic receptor 5-HT2B, overstimulates the glutamate release machinery [31, 32]. Inhibition of neuron-stimulated astroglial transcriptional activator κB-motif binding phosphoprotein (KBBP), and astroglial accumulation of reactive oxygen species (ROS) downregulates the astroglial glutamate reuptake transporter GLT1 [33]. At a symptomatic stage in sALS patients and transgenic SOD^1G93A^ mice, ‘synaptic stripping’ of the cholinergic connections from lower motor neurons (LMNs) to the inhibitory interneurons is concomitant with the loss of the feedback glycinergic and γ-aminobutyric acid (GABAergic) synapses from the inhibitory Renshaw cells to LMNs [34].

**Fig. (1):**
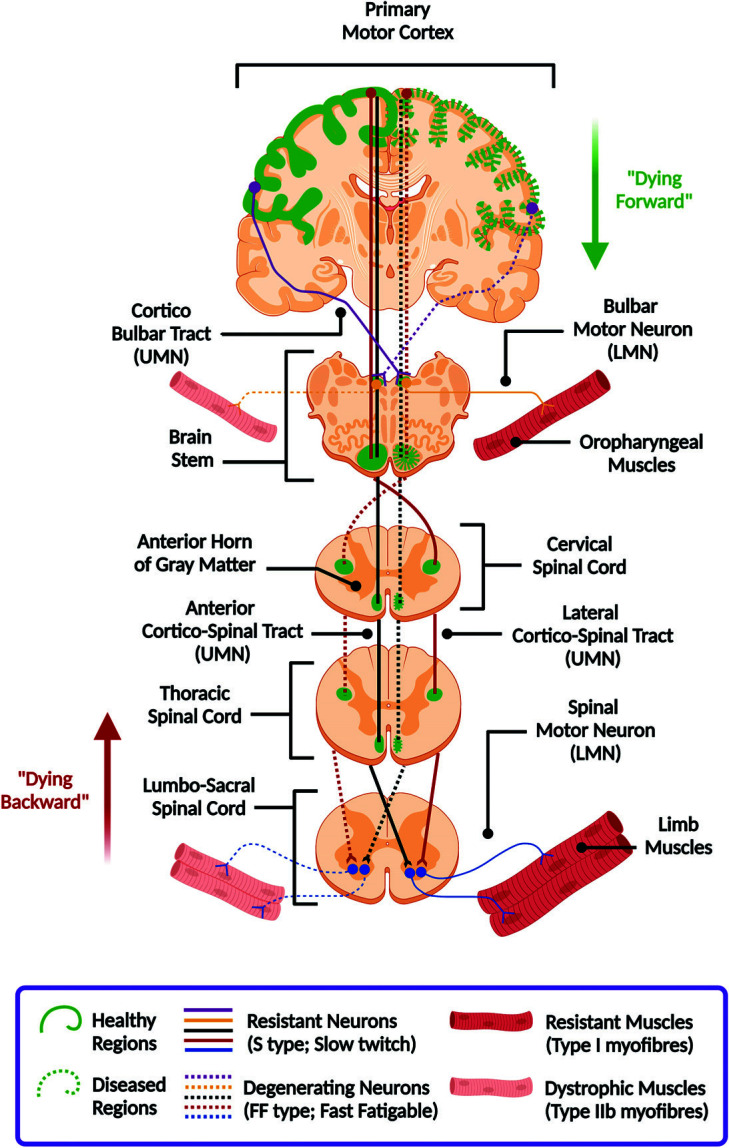
Pattern of the motor system dysfunction in ALS. Progressive degeneration affects the vulnerable fast-fatigable (FF) motor neurons and type IIb myofibres. Slow twitch (S) motor neurons and type I myofibres are resilient until the end stages. A ‘dying forward’ hypothesis proposes an anterograde degenerative trigger from cortex progresses towards spinal cord through trans-synaptic glutamate excitotoxicity. LMNs without a monosynaptic connection to corticomotor neurons are resilient to ALS pathology such as oculomotor, abducens, trochlear nerve, and Onuf's nuclei. A ‘dying back’ hypothesis suggests that an early retrograde degeneration trigger from neuromuscular junctions (NMJs) moves back towards primary motor cortex by distal axonopathy. Both UMN and LMN degeneration can also occur independently and in a stochastic manner during ALS progression, which might be attributed to oxidative stress mediated apoptosis. Reprinted from N Engl J Med 377:2, Brown, R.H.; Al-Chalabi, A., Amyotrophic Lateral Sclerosis, 162-172, Copyright (2017), with permission from (Massachusetts Medical Society).

Altogether, the gain of excitatory drive and loss of inhibitory regulation increases Ca^2+^ influx and triggers a sustained neuronal depolarization [35]. Due to the lack of Ca^2+^ -buffering proteins, defects in the Ca^2+^ ATPase pump and Ca^2+^/Na^+^ exchangers, and upregulation of voltage-gated Ca^2+^ channels, the neuronal hyperexcitability disrupts the intracellular Ca^2+^ homeostasis cycle across the endoplasmic reticulum (ER) and mitochondria [36, 37]. This leads to cellular ATP depletion and increased ROS production, which in turn triggers Na^+^/K^+^ ATPase pump dysfunction and further enhances neuronal firing [28]. Thus, the local motor circuitry is compromised to glutamate excitotoxicity. Notably, there are differences in opinion regarding the region of origin and the course of disease progression in ALS, influenced by the variable vulnerability of motor neurons and muscle fibre types (Fig. **1**).

#### 3.1.2 Effects on Neuromuscular Junctions

Mutant protein aggregates of SOD1 in skeletal muscles sequester cytoskeletal proteins like kinesin-associated protein (KAP3) [38]. Along with the downregulation of the acetylcholine transporter ChAT, this process inhibits acetylcholine (ACh) release from LMNs [38]. Overall, it results in the dismantling of neuromuscular junctions (NMJ) and a loss of myofibre contractility [39].

Release of neuronal growth-cone repellents like Nogo-A from skeletal muscles and chemorepellents like semaphorin-3A (SEMA3A) from dysfunctional terminal Schwann cells (TSCs) have been found to alter synaptic cytoskeletal dynamics by reducing phosphoglycerate kinase 1 (PGK1) production and by neuropilin (NRP) signaling, respectively [40, 41]. Concomitantly, various non-guidance factors from terminal Schwann cells and damaged myofibres alter the cell-cell adhesion networks [42, 43]. Along with an upregulation of denervation inducers or reinnervation inhibitors such as muscle-specific acetylcholine receptor subunits (AChRα1) or neuronal tyrosine kinases (ephrin type-A receptor 4, *i.e.*, EPHA4), these processes trigger distal axonopathy [44, 45]. Activation of aberrant acetylcholine receptors due to denervation (γ-AChR) and maladaptive reinnervation (ε-AChR) at the motor end plate disrupts the firing rate, connectivity, and regeneration potential of neurons [46]. This, in turn, enhances the fatigability of myofibres during spontaneous muscarinic activation [47]. Notably, axonal retraction at NMJs precedes neuronal death in SOD^1G93A^ mice and is in turn, preceded by a loss of TSCs [48, 49]. At the early stages of ALS, exhaustive rounds of regeneration and aberrant electrochemical activation deplete myogenic satellite cells [50].

#### 3.1.3 Selective Vulnerability of Motor Neuron and Skeletal Myofibre Subtypes

ALS-vulnerable fast-firing fast-fatigable (FF) motor neurons have larger cell bodies, faster axonal conduction velocity, elaborate dendritic branching, and high NMJ innervation as compared to the ALS-resistant slow-twitch fatigue-resistant (S-type) motor neurons [51]. FF motor neurons (MNs) innervate the fast-contracting extrafusal myofibres, while S-type neurons connect with the slow intrafusal fibres [37]. Moreover, ALS-vulnerable MNs are susceptible to oxidative stress and proteostasis dysregulation due to the low levels of free-radical scavengers and cytosolic calcium buffering proteins (*e.g.*, parvalbumin, calbindin, calreticulin) [52].

Overactivation of osteopontin-integrin-matrix metalloprotein 9 axis is involved in ER-stress-induced inflammation in FF neurons, with a concomitant upregulation of neurofilament proteins of retrograde transport. Moreover, upregulation of SIL1, the co-chaperone of ER-resident binding immunoglobulin protein (BiP), induces unfolded protein response (UPR); while the insulin growth factor (IGF-2/IGF-1) signalling protects against glutamate excitotoxicity in S-type neurons [37]. FF neurons express the Na^+^/K^+^ ATPase-α3 subunit that is selectively impaired by mutant proteins, while S-type neurons express the resilient Na^+^/K^+^ ATPase-α1 subunit [53]. Neuronal vulnerability is also dictated by the differential expression of GABAA-α2 and GLUR1 on FF neurons, with GABAA-α1 and GLUR2 on S-type neurons [54]. Notably, the apoptosis-inducing factor SEMA3A is secreted by the ALS-vulnerable type-IIb fast-twitch glycolytic myofibres, while the ALS-resistant type-I slow-twitch oxidative myofibres secrete SEMA3E [51]. Intriguingly, presynaptic Na^+^ channels (ENaC) can slow down the synaptic dysfunction by inducing a non-selective ‘presynaptic homeostatic plasticity’ at ALS-affected NMJs [55].

Although the dysfunction of motor circuitry is profound in ALS, there is evidence of sensory neuronal abnormalities across the heterogenous population of ALS patients. This enforces the idea of the multisystem nature of disease progression in ALS (details in **Box** **1**).

At presymptomatic stage, the synaptic dysfunction of fast fatigable α-MNs reduces the activity of fast contractile myofibres, and induces an increase in glutamatergic spindle-afferent inputs to α-MNs. This process triggers the excitotoxic death [60]. Hence, it is not clear whether hyperexcitability triggers neurodegeneration, or it arises to compensate neuronal death [61].

It is important to note that the differences in vulnerability of specific motor neuron or muscle fibre types potentially regulate the dominant heterogeneity in UMN and LMN involvement across various ALS phenotypes depending on the site of onset. A new line of ALS research is currently focused on targeting the unique structural, functional, and molecular features of these cell types for developing targeted preventive or therapeutic measures.

### 3.2 Non-cell Autonomous Neuroinflammation

Reactive microgliosis progresses in motor and even extra motor areas since the early presymptomatic stage [62]. It is preceded by reactive astrocytosis with regionally variable activation through inherent defects or paracrine signalling from neurons [63]. Given the dual role of astroglia in neuroprotection and neurotoxicity, a chronic disease state can drive an adaptive CNS-specific response towards systemic hyper-inflammation [64].

#### 3.2.1 Innate Immune Response

In the spinal cord of ALS patients and transgenic mouse models, cell-specific accumulation of mutant protein aggregates (SOD1, TDP43) induces the secretion of chemokines such as monocyte attractant MCP1 and B-cell attractant CXCL13 [65]. It also triggers the production of oxidative stress mediators such as ROS, inducible nitric oxide synthase (iNOS), cyclooxygenase 2 (COX2), and prostaglandin E2 (PE2) [66]. This, in turn, drives the production of pro-inflammatory cytokines such as TNF-α, interferon γ (IFN-γ), and interleukin 1β (IL-1β) [63]. Furthermore, it reduces the secretion of neurotrophic factors such as IGF-2, glial cell-derived neurotrophic factor (GDNF), brain-derived neurotrophic factor (BDNF), and vascular endothelial growth factor (VEGF) from microglia and astrocytes [67].

Microglia can be directly activated by chemokines or indirectly by mutant protein aggregates, excitotoxic stimuli, and extracellular ATP [68]. At a presymptomatic stage, the toxicity of mutant proteins in astrocytes induces Wnt/β-catenin signaling-dependent anti-inflammatory M2 microglial activation [69]. However, chronic NF-κB activation triggers reactive gliosis with inflammasome formation and blood-CNS barrier disruption [69]. Consequently, the pro-inflammatory M1 microglia secrete factors (TNF-α, IL-1α, C1q) to induce the neurotoxic A1 phenotype in astrocytes [70]. Together with the exosomal transport of astrocytic regulatory microRNAs and misfolded protein aggregates, the overexpression of astrocytic gap junction proteins (*e.g.*, connexin-43) facilitates the rapid spread of glial toxicity to the neighbouring cell types [71].

Despite the glial overexpression of the anti-inflammatory toll-like receptor (TLR2) at the presymptomatic stage, the activity of the pro-inflammatory receptor TLR4 in neurons induces a net neurotoxic environment [72]. At later stages, upregulation of high mobility group protein 1 (HMGB1), the inflammasome marker, exacerbates proinflammatory pathways through astroglial TLR4 signalling and microglial RAGE signalling [73, 74]. Altogether, these constitute the self-perpetuating cycle of microglial overactivation and neurodegeneration [62]. Additionally, astroglia mediates the infiltration of neutrophils and peripheral macrophages or monocytes through the secretion of specific chemoattractants such as MCP1, CXCL8, galectin-1, osteopontin, and macrophage colony-stimulating factor (M-CSF) [63]. Notably, variations in the 3’-untranslated region (3’-UTR) of the gene encoding interleukin-18 receptor accessory protein (IL18RAP) can regulate NF-κB signalling to prevent neurotoxicity of induced pluripotent stem cell (iPSC)-derived microglia from C9orf72 ALS patients [75].

#### 3.2.2 Adaptive Immune Response

At early stages, infiltration of CD4+ helper T-cells (Th) maintains a net neuroprotective niche through the secretion of anti-inflammatory cytokines (IL-4, transforming growth factor β, *i.e.*, TGF-β), and the stimulation of astrocytic GLT1 activity [76]. Downregulation of regulatory T-cells (Treg) and Th2 cells through the forkhead box P3 (FOXP3) and GATA-binding protein 3 (GATA3) transcription factors accelerates disease progression [77]. Moreover, Th17 and Th1 cells secrete proinflammatory cytokines (IL-17, IL-23, IL-10, IFN-γ) upon infiltration into the CNS microenvironment [78]. Together, these processes also upregulate the pro-inflammatory purinergic receptors P2X7 and P2Y12 in astroglia to induce inflammasome formation and caspase-1 activation [73, 79]. Activated CD14+ microglial cells acquire the characteristic surface markers of dendritic cells (CD11c, CD86, CD54), which promotes an adaptive immune response [63]. At later stages, cytotoxic CD8+ T-cells and natural killer cells infiltrate the CNS microenvironment through microglial pro-inflammatory TLR response and MN-specific antigen presentation [67, 78]. Additionally, diseased neurons secrete subunits of the complement system (*e.g.*, C1q, C3, C4, C5b-9) to relay the stress signalling to neighbouring cells [80]. Although the role of B-lymphocytes in ALS pathology is unclear, autoantibodies observed at end stages (*e.g.*, those against HMGB1, P/Q-type voltage-gated Ca^2+^ channel, AChR) indicate aberrant autoimmune activation [67].

#### 3.2.3 Role of Blood-CNS Barrier in Neuroinflammation

In medulla oblongata and spinal cord from sALS patients and transgenic SOD1 mice, the presence of dysfunctional microvessels indicates pervasive damage to the blood-brain barrier (BBB) and blood-spinal cord barrier (BSCB) with micro-haemorrhages [81]. This phenomenon hampers the recruitment of anti-inflammatory macrophages and Treg cells and induces a toxic neurovascular inflammatory response [81]. Ultrastructural analysis of the choroid plexus in ALS patients revealed the loss of tight junction components, disruption of vascular integrity, vascular injury, and loss of pericytes [82].

Currently, the initiation of immune response and the nature of the involvement of specific immune cells in ALS onset and progression have not been completely understood. This is because of the chronic nature and late diagnosis of ALS, which makes it difficult to determine whether abnormal immune response or inflammation is a cause or consequence of the disease [83].

### 3.3 Disrupted Neuron-astroglial Interactions

#### 3.3.1 Role in Pathogenic Processes

Disrupted intracellular Ca^2+^ signalling in astrocytes triggers the release of excitotoxic gliotransmitters (glutamate, D-serine) and proinflammatory factors that impair glutamate reuptake [71]. Additionally, astrocytic GLT1 expression is downregulated by NF-κB signalling through membralin depletion and metadherin upregulation [84, 85]. Upon cleavage by caspase-3 and the addition of small ubiquitin-like modifier (SUMO), accumulation of the GLT1 C-terminal fragment induces the reactive astrocytic phenotype [86]. Moreover, abnormal upregulation of GABA or glycine transporters (GAT1 or GlyT2) in gliosomes, with concomitant dysregulation of astrocytic cystine/glutamate antiporter (xCT) stimulates glutamate release from astrocytes and decreases the antioxidant glutathione levels [71]. Microglia-neuron interaction is also disrupted by the dysregulation of CD200/CD200R immune receptor signalling at the presymptomatic stage and CX3CL1/CX3CR1 chemokine receptor signalling at the symptomatic stage, respectively [87, 88]. Intriguingly, downregulation of the antigen presentation element MHC-1 by astrocytes triggers ER stress in neurons through proteasomal dysfunction, whereas enhanced TGF-β1 secretion by astrocytes dysregulates neuronal autophagy [89].

#### 3.3.2 Role in Loss of Neuroprotection

In ALS, there is a disruption of the metabolic coupling of spinal neurons with the astrocytes and oligodendrocytes. This results from the decreased transport of glycolytic products (lactate) for neuronal oxidative phosphorylation due to the loss of the astrocytic (MCT4) and oligodendrocytic (MCT1) lactate efflux transporters [90]. Together with the pro-nerve growth factor (pro-NGF) and p75 neurotrophin receptor (p75NTR) signalling in astrocytes during mitochondrial dysfunction, the neuroglial metabolic uncoupling triggers microglial activation [90]. There is a reduced secretion of astrocytic trophic factors that mediate neuroprotective cross-talk between astroglia and neurons such as BDNF, VEGF, NGF, plasminogen, basic fibroblast growth factor (bFGF), and neurotrophin 3/4 (NT-3/4) [91]. Death of oligodendrocytes reduces the secretion of trophic factors such as GDNF and ciliary neurotrophic factor (CNTF) to promote neurodegeneration [92] Interestingly, OPTN is known to inhibit NF-κB mediated astroglial activation, glutamate excitotoxicity, chronic inflammation, and necroptosis. Targeted depletion of OPTN in oligodendrocytes and microglia leads to reactive gliosis with demyelination and axonopathy [93]. Notably, microglial activation is facilitated by the necroptotic death of neurons and oligodendrocytes at early stages (details in **Box** **2**).

#### 3.3.3 Pathogenicity of Extracellular Biofluids

Chronic intracerebroventricular (i.c.v.) infusion of cerebrospinal fluid (CSF) from sALS patients into the transgenic mice expressing human TDP43 wild-type protein (hTDP43WT) induces cytoplasmic phosphorylated TDP43 aggregates, alteration of electrophysiological properties of corticomotor neurons, with subsequent motor impairment in mice [99]. Similar *in vitro* experiments trigger aberrant phosphorylation and aggregation of neurofilaments, reduced expression of ChAT and Na^+^/K^+^ ion pumps, reactive gliosis, and necroptosis of neurons [100]. Mass spectrometric analysis of CSF from sALS patients revealed an upregulation of inflammatory proteins (chitotriosidase, osteopontin, chitinase-3-like protein 1) and pro-inflammatory cytokines (TNF-α, IL-6), along with downregulation of anti-inflammatory cytokines (IL-10, IFN-γ) [101]. Additionally, there was a decrease in the levels of trophic factors (VEGF, GDNF) and an increase in neurotoxic mediators (PE2, COX2, ROS, NO, glutamate) [101].

Deletion of mutant SOD1 allele from motor neurons and oligodendrocytes in a transgenic mouse model of ALS delays disease onset but not progression [102, 103]. Mutant SOD1 depletion from microglia slows down disease progression by 50% but not the onset [104]. Targeted overexpression of mutant SOD1 in astrocytes and oligodendrocytes does not trigger the ALS phenotype. This highlights the need for distinct therapeutic interventions targeting the non-cell autonomous pathology at definite stages of the disease [105].

### 3.4 Dysregulation of Autophagy

Motor neurons (MNs) are terminally differentiated cells that heavily depend on constitutive autophagy for the clearance of misfolded protein aggregates, accumulated stress granules, damaged organelles, and defective cargo-loaded presynaptic vesicles [106]. Interestingly, autophagy displays a dual mode of action in ALS pathology. Activation of autophagy protects MNs and NMJs from an accumulation of mutant protein aggregates at the presymptomatic stage, whereas it mediates disease progression at the symptomatic stage. In spinal neurons and senescent skeletal muscles of ALS patients and mouse models, the accumulation of autophagosomes in the inclusion bodies at the symptomatic stage indicates an impairment of autophagy [107, 108]. Furthermore, transcription-independent accumulation of early-step autophagy factors (*e.g.*, Beclin-1, p62/SQSTM1, LC3-II, Atg5-Atg12 complex) is observed in motor neurons at the presymptomatic stage and in astroglia at later stages [109].

Over-induction of autophagy occurs as an adaptive response to a concomitant milieu of cellular stressors such as DNA damage stress, proteotoxic stress, ER stress, and oxidative stress. Increased autophagosome formation exceeds the lysosomal degradation rate and subsequently triggers apoptosis and type II cell death (autophagic-lysosomal) in various neurodegenerative disorders [110]. This process is associated with the downregulation of BDNF and its receptor tropomyosin receptor kinase B (TrkB) signalling, which otherwise protects neurons through the phosphatidylinositol-3-kinase/ protein kinase B/mammalian target of rapamycin (PI3K/ PKB/mTOR) pathway [111]. Age-associated impairment of lysosomal activity occurs due to the intra-lysosomal aggregates of lipofuscin granules, various extra-lysosomal aggregates, and damaged mitochondria. Altogether, these processes inhibit autophagy, which potentially contributes to the late symptoms [109].

#### 3.4.1 Autophagic Factors in ALS

The autophagic-lysosomal pathway can be disrupted by ALS-linked mutants of the core components or the modifier factors that regulate autophagic induction (C9ORF72, TBK1), autophagosome formation and maturation (OPTN, SQSTM1, TBK1, UBQLN2, VCP, VAPB), and autolysosome formation (C9ORF72, VCP, CHMP2B, ALS2) [109]. Loss-of-function C9orf72 mutations disrupt the transcription factor EB (TFEB) signalling of autophagic induction, impair the interaction with autophagy GTPases, and inhibit autolysosome formation [112, 113]. Repeat expansions from the gain-of-function C9orf72 mutations compromise the function of autophagy receptors like p62 and RNA-binding proteins like TDP43 by sequestering them in stress granules [114]. ALS-linked mutants of UBQLN2, p62, and OPTN impair the autophagic recognition of ubiquitinated aggresomes and damaged mitochondria [115]. TBK1 mutants impair the phosphorylation of autophagy receptors (p62, OPTN) and microtubule-binding proteins for cargo recognition and autolysosome formation [116].

Mutant SOD1 aggregates are known to inhibit mitophagy by sequestering OPTN, impair phagophore formation or expansion by binding the Beclin1-Bcl2 complex, and hinder autolysosome formation by sequestering the cytoskeletal dynein protein [109, 117]. Mutant TDP43 separately dysregulates mTOR-independent and mTOR-dependent autophagy by inhibiting the nuclear translocation of autophagy transcription factors (FOXO, TFEB), and by destabilizing the transcripts of autophagy factors (ATG7, DYNACTIN1, RAPTOR) [113, 118]. Mutant FUS dysregulates autophagy through the accumulation of autophagy receptors p62 and neighbour of BRCA1 gene 1 (NBR1) [119]. Furthermore, mutant HNRNPA1 is known to disrupt Beclin1 activity and stress granule dynamics to impair autophagic flux [120].

Notably, the autophagic machinery cross-talks with multiple cellular processes in order to regulate ALS pathogenesis. UBQLN2 can mediate the autophagy-independent degradation of aggresomes by proteasomal machinery, while it can exacerbate NF-κB mediated inflammatory response [121]. Furthermore, OPTN and p62 can regulate inflammatory response and necroptosis by interacting with TBK1 [116].

### 3.5 Blocked Axonal Transport

As polarized and elongated cells, motor neurons are susceptible to defects in endosomal trafficking and axonal transport due to the demand of a high turnover of membrane components, cargo-loaded vesicles, and mitochondria required for synaptic activity [122].

#### 3.5.1 Cytoskeletal Proteins in ALS

At early stages, mutant SOD1 impairs the anterograde transport in a cargo-specific manner, whereby dysfunctional mitochondria dissociate from the molecular motors of axonal transport [123]. Sequestration of the cytoskeletal dynein protein in stress granules (SG) by mutant SOD1 inhibits retrograde axonal transport and impairs the clearance of aggregates in neurons [124]. Loss-of-function C9ORF72 mutants disrupt the actin-mediated transport of lysosomal proteins and their association with early endosomes [125]. Loss-of-function OPTN mutants disrupt its interaction with myosin VI and impair the retrograde transport for autolysosome formation [126]. ALS-linked mutants of the tubulin-associated protein TUBA4A perturb the dynamics and stability of microtubules [127]. Mutants of profilin 1 (PFN1) disrupt its association with actin, while those in dynactin (DCTN) impair the formation of the microtubule-binding complex [109]. ALS-linked mutants of the p150Glued subunit of the dynein/dynactin complex also affect the binding to microtubules [128]. Notably, mutants of DCTN, PFN1, and TUBA4A are also known to dysregulate autophagy [109]. While the mutant forms of SOD1 and FUS can affect kinesins by altering their expression or phosphorylation state, the ALS-associated pathologic mutants of kinesin-5A have also been reported [109, 129].

#### 3.5.2 Neurofilaments in ALS

Upregulation of TNF-α perturbs the kinesin activity through p38 mitogen-activated protein kinase (MAPK) signalling, and impairs the anterograde transport [130]. Elevated glutamate levels impede the axonal transport of neurofilament heavy chain (NF-H) through phosphorylation [131]. Importantly, the accumulation of neurofilament light and medium chain proteins (NF-L, NF-M) along the axon acts as a pathological hallmark of ALS [109]. This is concomitant with the disruption of the neurofilament network and its reduced association with actin filaments and microtubules. Axonal transport defects at the presymptomatic stage impair synaptic transmission and exacerbate distal axonopathy [42].

### 3.6 Disruption of Endosomal Trafficking

Upon the disruption of the endosomal trafficking network, which cross-talks with autophagy and axonal transport, the viability of motor neurons (MNs) is critically impaired [122]. Cytoplasmic accumulation of mutant TDP43 disrupts the receptor signalling of cytokines to induce their mis-sorting towards early or recycling endosomes, which affects synaptic growth [132]. In iPSC-derived MNs from ALS patients carrying loss-of-function C9orf72 mutations, an increase has been observed in the transport of NMDA receptors and Ca^2+^ -permeable AMPA receptors to the surface of dendritic spines and neurites [125]. Moreover, defective transport of mannose-6-phosphate (M6P) receptors impairs the anterograde trafficking of lysosomal enzymes and cargo [125]. Due to the downregulation of vacuolar protein sorting VPS26, the endosomal retromer component, aberrant endosomal recycling has been reported for the BDNF/TrkB receptor [133]. These defects can arise from the disrupted interaction of C9ORF72 with Rab GTPases involved in the intracellular vesicle trafficking pathways [134].

**Fig. (4):**
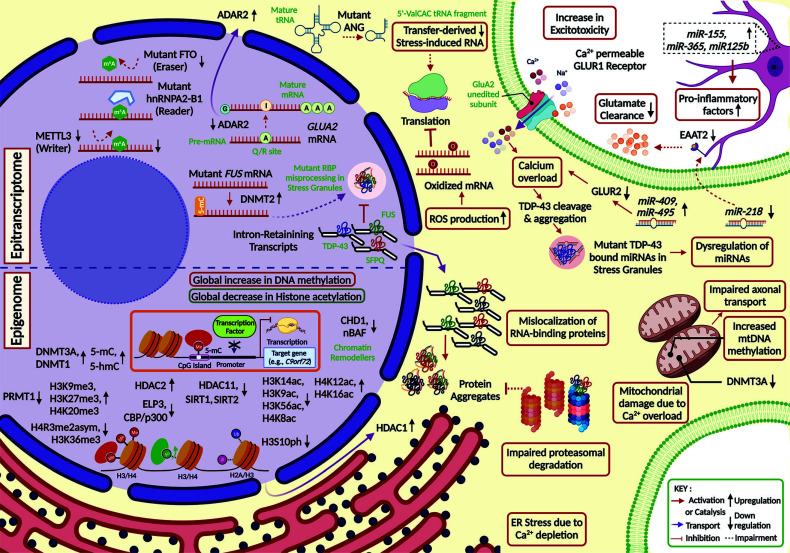
Changes in the epigenetic and epitranscriptomic landscape during ALS pathogenesis. ALS-associated epigenetic signatures include a global increase in DNA methylation with a concomitant deregulation of DNA methyltransferases (DNMTs). A global decrease in histone acetylation is concomitant with a dysregulation of histone acetyltransferases and histone deacetylases (HDACs). Global dysregulation of miRNA levels includes their sequestration in stress granules by mutant protein aggregates. ALS-relevant epitranscriptomic modifications commonly involves A-to-I editing defect in GluA2 transcript, which upregulates Ca^2+^ -permeable glutamate receptor in neurons. Accumulation of ROS-induced oxidized mRNA species, and tRNA fragment cleaved by mutant ANG impairs protein translation efficiency. Aberrant intron-retaining transcripts promote mislocalization of RNA binding proteins. Abbreviations: CHD2: chromodomain DNA helicase protein 2; CBP/p300: CREB binding protein p300; EAAT2: excitatory amino acid transporter 2; nBAF: neuronal Brg1-associated factor; PRMT1: protein arginine methyltransferase 1; SIRT: sirtuin; SFPQ: splice factor proline glutamine rich.

Similar to C9ORF72, alsin (ALS2) protein is a guanine nucleotide exchange factor (GEF) for the GTPase RAB5. Loss of ALS2 impairs endosomal mobility, increases the conversion of endosomes to lysosomes, and enhances the degradation of synaptic cargo like glutamate receptors [135]. Furthermore, dysregulated endosomal trafficking can be triggered by various rare mutants of VAPB, OPTN, CHMP2B, and the multivesicular body biogenesis factor ESCRTIII [7]. Mutants of VCP impair endocytosis through disruption of the clathrin-associated complex, impair disassembly and autophagic degradation of SGs, and can also trigger TDP43 mislocalization [136, 137]. Along with autophagic dysregulation, FIG4 variants with impaired phosphatase activity can perturb the cellular abundance of phosphatidylinositol 3,5-bisphosphate, a signalling lipid involved in the retrograde transport of endosomal vesicles [138]. Intriguingly, the defects in nucleo-cytoplasmic transport in ALS involve aberrant sequestration of its components in SGs, with dysfunctional RAN GTPase activity [139]. FUS mutants disrupt the transportin-mediated nuclear import, whereas the nuclear egress of TDP43 and FUS mutants occurs independently of exportin-1 [140]. Fig. (**2**) provides an overview of the dysregulated intracellular pathogenic processes across the neuron-glia network affected in ALS.

### 3.7 Dysregulation of RNA Metabolism and Protein Homeostasis Machinery

Motor neurons (MNs) and skeletal muscles are known to bear a metastable sub-proteome, which is characterized by the super-saturated levels of aggregation-prone proteins [141]. It is majorly due to the relatively low expression of chaperones and components of the ubiquitin proteasomal system (UPS), the protein quality control machinery mechanistically integrated with autophagy [142]. ALS-linked mutants of the aggregation-prone RNA-binding proteins (TDP43, FUS, SOD1) and autophagy factors (UBQLN2, OPTN, SQSTM1) aggravate their misfolding to form aggregates and fibrils [143]. Notably, ALS-vulnerable neurons and muscles suffer from excessive or impaired activation of both UPS and autophagy [144, 145]. Cytoplasmic aggregates of ALS-linked mutant proteins act as the ‘nucleation centers’ for subsequent misfolding and aggregation of native proteins that results in their loss of function [143]. Moreover, this generates the compact poly-ubiquitinated inclusion bodies that act as the immuno-histopathological hallmarks of ALS cases.

#### 3.7.1 Hallmark Inclusion Bodies

Ubiquitinated TDP43 aggregates are commonly present in 97% of cases of ALS and 50% of cases of ALS-FTD [146]. These form hyper-phosphorylated skein-like inclusion bodies in the frontal cortex, brain stem, spinal cord, and hippocampus of TARDBP fALS or fALS-FTD and sALS cases [146]. Mutant SOD1 aggregates are known to form fibrillated Lewy bodies and hyaline conglomerate inclusion bodies in SOD1 fALS and sALS cases [147]. FUS aggregates produce basophilic tangle-like inclusion bodies (Bunina bodies) in the spinal cord, motor cortex, and hippocampus of FUS fALS and sALS cases [148]. Interestingly, TDP43 or FUS positive inclusions are not observed in SOD1 fALS cases, although misfolding of SOD1 is triggered in both FUS fALS and TARDBP fALS cases [149]. This suggests a divergent mechanism of misfolding or aggregation across various proteinopathies. Misfolded OPTN and UBQLN2 form skein-like bodies in the frontal cortex and spinal cord in sALS cases and are also found to colocalize with TDP43 and FUS in stress granules [126, 150]. C9ORF72 dipeptide repeats, mainly poly(GA), form ubiquitin-positive bodies in neurons and muscles of C9orf72 fALS-FTD and sALS cases [151]. This can be present with wild-type or misfolded TDP43 aggregates in the cortex and spinal cord or p62 aggregates in the hippocampus and cerebellum [151].

Overexpression or mutation of neurofilament chain proteins, dynein, peripherin, and microtubule-associated protein tau MAPT (reported in ALS-dementia-parkinsonism syndrome) have been found to induce neurofilament aggregation in ALS [7, 152]. Glutamate-induced overexpression of kinases (MAPK, PIN1) induces hyperphosphorylation of neurofilaments that leads to the formation of large perikaryal inclusion bodies and disruption of axonal circuitry [152]. Altogether, the aggregates of misfolded proteins and neurofilaments enforce the global failure of proteasomal and autophagic degradation machinery in ALS.

#### 3.7.2 Dysfunction of Ubiquitin Proteasomal System

In the early stages of ALS, a significant impairment of both total and specific catalytic activity of the ubiquitin proteasomal system (UPS) is usually observed [153]. Furthermore, overexpression of the inducible proteasome subunits occurs early during disease progression, which is followed by the downregulation of the constitutive proteasome subunits [153]. This process is associated with a local inflammatory response mediated by reactive gliosis [154]. While the expression of 26S proteasome is unperturbed in SOD^1G93A^ mice, the levels of 20S proteasome components are specifically decreased in motor neurons of the lumbar spinal cord [155]. Intriguingly, an upregulation of UPS activity underlies the sustained clearance of toxic protein aggregates in the skeletal muscles of SOD^1G93A^ mice. However, excessive activation of UPS results in muscle atrophy through the degradation of myofibrillar proteins and inhibition of myogenesis [156].

Aggregation-prone C9ORF72 dipeptide repeats are known to trigger neurotoxicity by impairing proteasomal activity [151]. ALS-linked mutants of VCP disrupt its interaction with the proteasome and thus impair the proteolysis of bound ubiquitylated substrates [157]. ALS-linked mutants of UBQLN2 induce the accumulation of UBQLN2-positive inclusions, which impair the cargo delivery to the proteasome [158]. Moreover, the downregulation of the proteasomal components triggers the mislocalization and aggregation of ubiquitylated proteins and therefore has been associated with the formation of histopathological marks in ALS [143]. Mutants of cyclin F (CCNF), a component of the E3-ubiquitin ligase complex, induce abnormal accumulation of TDP43 in both fALS and sALS cases [159].

#### 3.7.3 Dysregulation of RNA Metabolism

RNA metabolism and protein synthesis are intricately linked through the activity of dynamic stress granules, and thus there is a concomitant dysregulation of both processes in ALS [160, 161]. ALS-linked mutants of nuclear RNA-binding proteins TDP43, FUS, hnRNPA1, hnRNPA2B1, ATXN2, and matrin 3 (MATR3) form mislocalized cytoplasmic aggregates because of the irreversible interactions with their prion-like low-complexity domains [162]. This alters the assembly and disassembly of ribonucleoprotein granules (SG, P-bodies) that act as nucleation centers for native protein aggregation [161].

By binding to the 3'-UTR of mRNAs, both TDP43 and FUS regulate presynaptic mRNA transport, mRNA splicing, and protein turnover [146, 163]. They also regulate miRNA processing and transport *via* interaction with the Drosha complex, stabilize long non-coding RNAs, and mediate transcriptional repression of long UG-rich intronic sequences [163]. Mutations in TARDBP occur commonly in the domains involved in protein-protein interaction and nuclear transport, which induces dysregulation of the target transcripts by sequestration in mRNA-silencing foci [164]. These transcripts usually encode RNA-binding proteins (TDP43, FUS, VCP), translation initiation factors (eukaryotic initiation factor EIF2α), microtubule-stabilizing factors (microtubule-associated protein MAP1B), constitutive heat-shock chaperones (heat shock 70 kDa protein cognate 4, *i.e.*, HSC70-4), and neuronal survival proteins (progranulin, choline acetyltransferase) [165].

ALS-linked mutations in FUS occur mostly in the domains carrying the nuclear localization signal and intrinsically disordered regions. Similar to TDP43, such mutants mediate the cytoplasmic mislocalization of FUS [165]. Aggregation of mutant FUS with nucleo-cytoplasmic shuttling proteins (HNRNPA1, HNRNPA2B1), spliceosome assembling proteins (SMN1), and mRNA processing complexes has been found to promote the dysregulation of translatome [166]. Notably, C9orf72 hexanucleotide repeat expansions (HRE) induce defects in nucleo-cytoplasmic shuttling, post-transcriptional processing, ribosomal biogenesis, and assembly of stress granules [160]. RNA foci with transcribed G4C2 repeats of C9orf72 can sequester other RNA-binding proteins [167]. Repeat associated non-AUG (RAN) mediated translation of these transcripts and their anti-sense mRNA can produce toxic dipeptide repeats (DPRs) [168]. Among these DPRs, poly(GA) mainly induces protein aggregation by impairing UPS to trigger ER stress, while poly(GR) and poly(PR) can inhibit global translation by sequestering RNA-binding proteins (FUS, TDP43, ribosomal biogenesis factors) along with their bound transcripts inside stress granules [168].

ALS-linked mutants of the ribonuclease ANG disrupt rRNA transcription and tRNA processing, whereas mutants of the RNA helicase SETX destabilize the transcriptional RNP complexes [7]. Moreover, mutants of other RNA-binding proteins ATXN2, HNRNPA1, HNRNPA2B1, TATA-binding protein associated factor 15 (TAF15), and EWS RNA-binding protein 1 (EWSR1) have been directly associated with ALS pathology [160]. In OPTNE478G fALS cases, the proteotoxic profile resembles that of other proteinopathies involving phosphorylated TDP43, tau, and α-synuclein [169].

Notably, it is a widespread idea in the field that the spread of misfolding and toxic protein aggregates might underlie the course of disease progression (details in **Box** **3**). Development of sensitive assays for the early detection of ALS-relevant misfolded proteins would allow necessary interventions for clearance before they accumulate into large degradation-resistant aggregates and thus prevent the pathological cascade of proteotoxicity.

### 3.8 Defective DNA Damage Repair System

Defective or toxic DNA damage signalling and repair (DDR), accumulation of endogenous genotoxic stressors (such as ROS), and chromatin disorganization can collectively trigger genomic instability that causes irreversible damage to the terminally differentiated motor neurons. ALS-linked mutants of various cell cycle regulator genes (NEK1, C21ORF2, CCNF) are known to induce abnormalities in DNA damage repair [172]. Mutants of VCP impair DNA damage response through the loss of interaction with non-homologous end-joining repair (NHEJ) factors Ku70/80 and ring finger protein 168 (RNF168) [172]. These defects can be exacerbated by mutants of SQSTM1 through the loss of interaction with other NHEJ factors, such as ataxia telangiectasia mutated (ATM) and RAD50 [172, 173]. In ALS/FTD cases, cytoplasmic mislocalization of TDP43 impairs the nuclear transport of the DNA repair complex of X-ray repair cross-complementing complex 4 (XRCC4) and DNA ligase IV (LIG4), which results in toxic accumulation of DNA double-strand breaks (DSBs) [174]. Additionally, mutant FUS impairs the recruitment of the DNA repair complex XRCC1/LIG3 to DNA single-strand breaks (SSBs) following oxidative damage, which impairs the base excision repair (BER) [175].

Intriguingly, mutant TDP43 and FUS dysregulate DNA: RNA hybrid (R-loop) formation and thus promote transcriptional stress [176]. Transcription of C9orf72 repeat expansions facilitates the accumulation of R-loops as well as the formation of toxic DNA secondary structures, hairpins, and G-quadruplexes [177]. C9orf72 RNA repeat expansion (RRE) foci sequester various RNA‐binding proteins (TDP43, FUS, NPM1, APE1), and thus impairs their DNA damage repair activity [178]. Moreover, dipeptide repeats secreted by C9orf72 fALS astrocytes can trigger genomic instability by sequestering DNA repair factors, inducing R-loop formation, and chromatin compaction [179]. ALS-linked mutants of the R-loop processing factors such as SETX, ATXN2, and HNRNPD are well known [180]. Furthermore, neuronal accumulation of C9ORF72 dipeptide repeats at the nuclear membrane can trigger chromatin disorganization [181]. In both fALS and FTD cases, TBK1 haploinsufficiency leads to chromosomal instability. Cytoplasmic accumulation of ssDNA in ALS-affected neurons over-activates the cyclic GMP/ AMP synthase (cGAS) and stimulator of interferon gene (STING) signaling to trigger a proinflammatory response [182]. Mutants of other ALS-relevant proteins (MATR3, ERBB4), as well as disease modifiers like histone acetyltransferase ELP3, mRNA export factor GLE1, and stress granule component TIA1, have also been found to deregulate DNA damage repair [172].

### 3.9 Dyshomeostasis of Endoplasmic Reticulum-Mitochondria Functional Networks

Due to the continuous firing of ALS-vulnerable motor neurons (MNs), the chronic depletion of Ca^2+^ reserves in the endoplasmic reticulum (ER) leads to ER stress [183]. Concurrently, the chronic Ca^2+^ overload in mitochondria activates pro-apoptotic factors to trigger the release of cell death signals [183]. Loss-of-function mutations in TARDBP and FUS are known to impair autophagosome formation by interfering with ER-Golgi transport and ER-mitochondria interactions. These proteins also compromise mitophagy through the sequestration of the E3 ubiquitin ligase PARKIN [184]. Notably, the dysfunction of selective autophagy contributes to RNA metabolism dysregulation, ER stress, and mitochondrial damage in motor neurons [185]. Accumulation of ROS further inhibits autophagy, stabilises misfolded protein aggregates, and promotes inflammation. This entire loop exacerbates ER stress and mitochondrial damage that results in apoptosis [186].

#### 3.9.1 ER-associated Dysfunction

Astrocytic deletion of membralin, a factor involved in ER-associated degradation (ERAD), facilitates neuronal excitotoxicity [84]. Mutant SOD1 is known to dysregulate the stress granule formation by colocalizing with its core components like RasGTPase-activating protein-binding protein 1 (G3BP1) and T-cell intracellular antigen 1 (TIA1) [187]. At the early symptomatic stage, a selective induction of the PRKR-like ER kinase (PERK) arm of neuronal unfolded protein response (UPR) has been reported [188]. However, this fails to prevent misfolding of mutant proteins due to the naturally low levels of ER-resident chaperones. In transgenic SOD1 mice and sALS patients, the UPR machinery upregulates protein disulphide isomerase (PDI) and other ER-resident chaperones (protein disulphide isomerase PDIA3) in astrocytes and oligodendrocytes [189]. Furthermore, inactive proteins sequestered in stress granules suppress the general translation process and ERAD pathways [190]. Aggregation of mutant VAPB, an integral ER membrane protein, dysregulates the activating transcription factor 6 (ATF6) and X-box binding protein 1 (XBP1) arms of UPR [191]. Despite being an initial adaptive response to ER stress, the prolonged activation of UPR leads to apoptosis [188].

#### 3.9.2 Golgi-associated Dysfunction

ALS-associated mutant proteins can induce Golgi fragmentation by the inhibition of vesicular trafficking in the ER-Golgi and Golgi-plasma membrane networks [192]. Given that the axonal Golgi networks regulate the local synthesis and trafficking of axonal membrane proteins, the Golgi fragmentation can disrupt axonal secretory transport and autophagy [192]. Notably, the fragmentation of somatic and dendritic Golgi precedes axonal retraction and muscle denervation in ALS mouse models [193].

#### 3.9.3 Mitochondrial Dysfunction

In ALS patients, Ca^2+^ homeostasis is disrupted in the distal dendrites and axons due to the block of mitochondrial transport, with concurrent mitochondrial accumulation in the soma and proximal axon hillock [194]. MNs derived from the spinal cord of ALS patients and presymptomatic SOD^1G93A^ mice show mutant SOD1 aggregates in intermembrane space, mitochondrial swelling, fragmentation of the mitochondrial network, peroxidation of mitochondrial membrane lipids, decreased coupling between mitochondrial oxidative phosphorylation and electron transport chain (ETC), and reduced expression of mitochondrial enzymes [195]. Downregulation of peroxisome proliferator-activated receptor gamma coactivator 1α (PGC-1α), a master regulator of mitochondrial biogenesis and function, exacerbates disease progression across CNS and skeletal muscles of ALS [196]. At the disease onset, dense clusters of presynaptic mitochondria are observed in spinal neurons due to the downregulation of mitochondrial transporter MIRO1 [197]. Moreover, TDP43 inclusion bodies interact with critical mediators of mitochondrial dynamics and mitophagy, such as prohibitin 2 (PHB2) [198]. ALS-linked mutants of VAPB and VCP disrupt the mitochondrial-ER contacts (MERCs) to trigger organellar dysfunction [199]. Dysfunction of mitochondrial complex I and complex V subunits of ETC has been observed in C9orf72 ALS [200].

### 3.10 Oxidative Stress

In the astrocytes derived from ALS patients and mouse models, there is a decrease in the nuclear factor erythroid 2-related factor 2 (Nrf2)-dependent antioxidant signalling. This is associated with the depletion of glutathione, upregulation of peroxiredoxins and catalase activity, and persistent microglial inflammatory damage to the neighbouring neurons [201]. In the motor cortex, depletion of mitochondrial glutathione reductase (GSH) potentially arises from sustained glutamatergic signalling, and in turn, facilitates mitochondrial Ca^2+^ release. This leads to dysfunction of the voltage‐dependent anion channel (VDAC) and adenine nucleotide translocator (ANT), along with the opening of apoptotic mitochondrial permeability transition pore (mtPTP) channel in ALS-affected neurons [202]. Mitochondrial ROS accumulation induces glial glutamate release by the upregulation of cysteine-glutamate antiporters, which further promotes oxidative stress due to decreased cysteine uptake [203]. Furthermore, the presynaptic decline at NMJs is facilitated by ROS released by the glial cells and infiltrated immune cells [204]. Cytoplasmic mislocalization of mutant SOD1 impedes the transcription of genes involved in DNA damage response and antioxidant pathways [205]. Mutant TDP43 induces oxidative stress through the nuclear accumulation of Nrf2 and activation of mitochondrial UPR machinery. In turn, oxidative stress promotes GADD34-mediated phosphorylation as well as aggregation of acetylated TDP43 [206]. Mutant FUS inhibits the repair of oxidative stress-mediated DNA damage through defects in DNA ligation [201]. Similar to the ALS-linked mutants of D-amino acid oxidase (DAO) and ALS2 proteins, the toxic dipeptide repeats of C9ORF72 promote ROS generation [172]. Understandably, the exogenous pro-oxidative stress factors such as intense physical activity, radiation, metal toxicity etc., as well as endogenous modifiers such as malnutrition, cachexia, respiratory weakness etc., can exacerbate the disease progression in ALS [207].

#### 3.11 Dysregulation of Metabolic Programming

Rapid cycles of action potential propagation and presynaptic transport across the long axon of fast-firing motor neurons are major factors for the high energy demand of CNS [37, 208]. Moreover, high ATP levels are required to prevent the aggregate formation in both soma and axon [209]. Neuronal energy homeostasis is linked with the antioxidant glutathione machinery through a fine balance between the glycolytic and pentose phosphate pathway (PPP) flux [210]. High metabolic demand and low oxidative stress response of corticospinal neurons and skeletal muscles render them selectively vulnerable to the energy defects in ALS. This impairs self-regenerative pathways, which facilitates terminal nerve retraction and loss of reinnervation [211]. In order to produce *in situ* energy substrates during impaired neuronal glucose uptake, ROS generated by the mitochondrial ETC system stimulates lipid transport to astrocytes, which otherwise accelerates neurodegeneration [212].

In ALS patients with pure UMN involvement, positron emission tomography (PET) imaging using 2-deoxy-2-fluoro-D-glucose (18FDG) revealed glucose hypo-metabolism across the fronto-dorsolateral cortex, precentral cortex, and temporal cortex [213]. However, a diverse glucose hyper-metabolism pattern is found in the midbrain, spinal cord, and skeletal muscles of ALS patients [214, 215]. Severe metabolic impairment occurs in the precentral gyrus during the bulbar onset and in the cervical cord during the upper limb onset [214]. Therefore, diverse states of metabolic pathogenicity occur spatiotemporally across CNS during the disease progression. In SOD^1G93A^ mice, glucose uptake in the spinal cord increases at the presymptomatic stage, but it declines progressively [216]. In the spinal cord of end-stage ALS patients and SOD^1G93A^ mice, the uncoupling of blood flow and glucose metabolism is observed with a concomitant increase in glycogen storage [217]. There is a persistent downregulation of glycolysis rate-limiting enzyme phosphofructokinase 1 (PFK1), Kreb's cycle enzyme succinate dehydrogenase (SDH), and Kreb's cycle intermediates in the CNS and skeletal muscles [218, 219]. Interestingly, impaired neuronal glucose oxidation has been associated with unperturbed astrocytic Kreb’s cycle at symptomatic stages in SOD^1G93A^ mice [220]. Dysregulation of nucleotide metabolism and histidine metabolism during ALS progression might serve as a metabolic signature of pervasive DNA damage by oxidative stress [180].

#### 3.11.1 ‘Hyper’ Metabolism in ALS

Systemic hypermetabolism, accompanied by dyslipidaemia, has been reported at the presymptomatic stage in ALS patients and SOD^1G93A^ mice [221]. The shift from glucose-dependent to lipid-based metabolism may be an adaptive response against the disrupted energy homeostasis at early stages [222]. Ketosis in astroglia promotes the reuptake of extracellular glutamate and induces the synthesis of GABA from glutamine in neurons [223]. Notably, endogenous energy reserves of skeletal muscles and adipose tissues are progressively depleted during the course of the disease. As a result, ALS patients often develop insulin resistance due to the dysregulated glycogen metabolism in muscles [224]. Proteomic analysis of motor neurons from SODG93A mice revealed the upregulation of hydroxy acyl-CoA dehydrogenase subunit A (HADHA) and acetyl-CoA acetyltransferase 2 (ACAT2) enzymes involved in ketosis, and β-hydroxy β-methyl glutaryl-CoA (HMG-CoA synthase) involved in cholesterol metabolism [225]. Interestingly, lipid accumulation during ALS progression is independent of the downregulation of enzymatic and transcriptional regulators of lipid synthesis [226].

In the spinal cord of transgenic SOD1G86R mouse, inhibition of β-glucocerebrosidase is found to promote glycosphingolipid metabolism, which delays ALS onset by inducing muscle reinnervation [227]. Interestingly, the ganglioside GM1a interacts with the BDNF receptor TrkA and with the Na^+^/Ca^2+^ exchanger at the nuclear membrane to facilitate Ca^2+^ transfer to ER [228]. Towards later stages, prolonged lipid hypermetabolism promotes oxidative stress *via* the by-products of ketosis and cholesterol synthesis in the spinal cord and skeletal muscles [226]. Notably, the progressive decrease of membrane fluidity observed in ALS mouse models can be attributed to the increased peroxidation of polyunsaturated fatty acids, which generates excitotoxic intermediates like 4-hydroxynonenal, and inflammatory mediators like eicosanoids [229]. Moreover, the excessive production of cholesterol and sphingolipids impairs vesicular transport, neurotransmitter cycling, and cytoskeletal organization and triggers excitotoxicity through defective RNA editing of GLUR2 transcript in neurons [230]. This process disrupts the ‘cholesterol sensor’ LXR signalling, which induces apoptosis [231].

Multiple pilot clinical trials have suggested that a high-fat diet can improve prognosis and delay disease progression in ALS patients by promoting LDL/HDL ratios or body mass index [232]. Importantly, approaches that target various steps of the neuroglial glucose metabolism might have potential in ALS therapy [218]. Fig. (**3**) provides an overview of the non-cell autonomous and cross-talking pathogenic processes across the neuroglial networks and neuromuscular junctions affected in ALS.

## 4 GENE & ENVIRONMENT: EPIGENETIC & EPITRANSCRIPTOMIC LANDSCAPE IN ALS

Only ~30 environmental risk factors have been significantly associated with ALS incidence till date through summary statistics from genome-wide association studies, linkage disequilibrium score regression, and Mendelian randomization analyses [16]. These factors include intense physical activity, psychiatric disorders, military service, contact sports, comorbid diseases (Type I diabetes, viral infections), and exposure to neurotoxins, heavy metals and organic solvents [233]. As per the gene-environment-time interaction (GETI) hypothesis of ALS incidence, a genetic risk variant can interact with a combination of environmental risk factors at a specific age in a particular geographical region for a definite period of time [16]. A panel of inherited genetic defects with varying penetrance might differentially trigger the primary pathogenic pathway. It needs to be complemented by variable intensities and combinations of environmental triggers to affect the secondary pathways that cumulatively set off the pathological cascade for ALS onset [234].

Epigenetic modifications involve the reversible heritable or non-heritable alterations in gene expression without any changes to the genome sequence, in response to endogenous and environmental factors [235]. Changes in the epitranscriptome (RNA epigenetics) reveal a novel code of locus and cell type-specific modifications on different classes of RNA for a dynamic and rapid co-transcriptional and post-transcriptional regulation of RNA expression, stability, localization, and function [236].

### 4.1 Epigenetic Signatures in ALS

In spinal LMNs of sALS and C9orf72 fALS patients, immunohistochemical studies revealed an increase in methylation and hydroxymethylation of CpG islands at gene promoters, which inversely correlated with TDP43 pathology [237]. Quantitative analyses of methylation profiles depicted that 5’-hydroxymethylcytosine (5hmC) levels are increased in corticospinal motor neurons of hSOD^1G93A^ mice and decreased in prpTDP43A315T mice [238]. Repeat-primed and methylation-specific PCR revealed hypermethylation of CpG islands near the C9orf72 repeat expansions in blood and brain samples from 10-30% of ALS and FTD cases [239]. Notably, increased 5’-methylcytosine (5mC) levels in mitochondria (especially at 16S rRNA gene) and downregulation of DNA methyltransferase DNMT3A, associated with mitophagy, were reported in skeletal muscles and spinal motor neurons of transgenic SOD1 mice [240].

In ALS patients, deficiency of the histone acetyltransferase (HAT) elongator acetyltransferase complex subunit 3 (ELP3) leads to a decrease in the transcriptional activation acetylation marks on lysine 14 of histone H3 (H3K14ac) and lysine 8 of histone H4 (H4K8ac) [241]. In SOD^1G93A^ mice, inhibition of the histone deacetylase HDAC6 at early stages increases α-tubulin and TDP43 acetylation, while HDAC6 overexpression at late stages promotes autophagy [242, 243]. In skeletal muscles of SOD^1G93A^ mice and ALS patients with rapid disease progression, HDAC4 is upregulated at presymptomatic stages and protects from skeletal muscle atrophy but is decreased upon symptom onset [244]. In primary cultures of spinal motor neurons of transgenic FUSR495X mice, downregulation of protein arginine N-methyl transferase 1 (PRMT1) is associated with a decrease in asymmetric di-methylation on arginine 3 of histone H4 (H4R3me2asym), with concomitant downregulation of H3K9ac and H3K14ac marks [245]. ALS-linked TDP43M337V and FUSR495X mutants have been found to reduce the global levels of conjugate H3 phosphorylation at serine 10 and acetylation at lysine 14 (H3S10Ph-K14Ac), which in turn dysregulates NF-κB signalling [246]. Distinct alteration patterns of the cross-talking histone methylation and histone acetylation marks suggest that each proteinopathy dictates its distinct histone modification landscape to converge on the pathological hallmarks of ALS.

Despite a wide variety of studies regarding miRNA deregulation in ALS patients, there is a limited overlap of the miRNA signatures, possibly because of the variation in study populations, types of control subjects, disease stages, sample sources, methods of tissue extraction, and miRNA expression profiling [247]. A recent meta-analysis of all available studies on miRNA dysregulation in ALS has identified four miRNAs (hsa-miR-9-5p/3p, hsa-miR-28-5p/3p, hsa-miR-132-5p/3p, hsa-miR-146a-5p/3p) to be the most commonly reported across nervous tissue, cerebrospinal fluid, skeletal muscles, and plasma or blood or serum [248]. A competitive endogenous circRNA-miRNA-mRNA axis reported in ALS involves the circRNA hsa-circ-0023919 that inhibits miR-9, which otherwise regulates the transcription of intermediate filament NEFL and leads to neurofilament aggregation [249].

### 4.2 Epitranscriptomic Landscape of ALS

Molecular heterogeneity in ALS etiopathogenesis can potentially be attributed to aberrant RNA modifications on various RNA substrates or a modification-independent function of the RNA modifiers [250]. In the spinal cord of ALS patients and transgenic SOD1 mice, accumulation of oxidized mRNA species bearing 8-oxo-7,8-dihydroguanosine (8OHG) has been consistently observed [251]. Oxidation of mRNA species has been primarily found in motor neurons and oligodendrocytes at the early presymptomatic stage, before the oxidation of lipids, proteins, and DNA during the symptomatic stage [251]. Post-transcriptional adenine-to-inosine (A-to-I) RNA editing at the glutamine/arginine (Q/R) site in GluA2, a subunit of glutamate receptor GluR2, is essential to prevent excitotoxicity in spinal motor neurons of sALS patients [252]. Upon conditional knockout of the RNA editing enzyme adenosine deaminase acting on RNA 2 (ADAR2) in mice, the motor neurons expressing Q/R site-unedited GluA2 subunit undergo apoptosis due to calcium overload and dysregulated autophagy [253]. The mislocalization of ADAR2 has been reported in the motor cortex and lumbar spinal cord of C9orf72 ALS/FTD patients [254].

Through the technique of methylated RNA immunoprecipitation with sequencing (MeRIP-seq), a global decrease in the level of N6-methyladenosine (m6A) modification on RNA has been observed upon TARDBP knockdown in mammalian cells [255]. This phenomenon is similar to that observed upon silencing of the m6A writer METTL3, which regulates synaptic growth at neuromuscular junctions [255]. Interestingly, mutations in the m6A reader hnRNPA2B1, which also regulates proteostasis, have been frequently associated with ALS [256]. Whole-genome sequencing revealed a positive association of motor neuron-specific variants in fat mass and obesity-associated protein (FTO), an m6A eraser factor from the α-ketoglutarate dependent dioxygenase family, with sALS cases [257]. Upon the loss of site-specific m5C modifications during cellular stress, the ALS-associated ribonuclease ANG promotes the cleavage of tRNAs to generate tRNA-derived small RNAs (tsRNAs) and triggers the assembly of stress granules [258]. Deregulation of the RNA methyltransferase DNMT2, which otherwise prevents tsRNA accumulation has been observed in FUS fALS cases with a concomitant increase in 5mC mark on the proximal FUS promoter, a lower FUS expression and a higher cytoplasmic FUS aggregation in motor neurons [259]. Notably, accumulation of 5’ValCAC tRNA fragment (valine tRNA with CAC anticodon) with increased ANG expression has been reported in the spinal cord and serum from SOD^1G93A^ mice at the symptom onset [260]. A recently discovered hallmark of ALS-FTD cases involves the nuclear depletion and cytoplasmic accumulation of the neuronal-enriched splicing factor SFPQ, which produces aberrant intron-retaining (IR) transcripts of neuronal proteins for accumulation in RNA granules at neurites [261]. A broad overview of commonly reported epigenetic and epitranscriptomic signatures of ALS pathogenesis is given in Fig. (**4**).

## 5 CHALLENGES AND PROSPECTS FOR ALS PATHOMECHANISTIC RESEARCH

The Food and Drug Administration (FDA) has currently approved very few drugs, namely Riluzole, Edaravone, and NueDexta, that can only moderately improve survival across specific ALS patient populations. Widespread efforts for treatments targeting the pathomechanistic networks of ALS have led to several late-stage clinical trials, with the primary therapeutic target being neuroinflammatory pathways (particularly the complement system) followed by oxidative and proteotoxic stress response [262]. These involve inhibitor molecules, genetic therapies through antisense oligonucleotides (ASO), stem-cell-based therapies, and recombinant humanized monoclonal antibodies [263]. Notably, the tyrosine kinase inhibitor Masitinib has been reported to prolong survival in phase III clinical trials by targeting macrophages, mast cells, and microglia [264]. For a detailed review of previously tested and failed treatment strategies, and novel therapeutics (including the repurposing of available drugs) in the pipeline, the readers can refer to Corcia *et al.* 2021 [263] and Mead *et al.* 2023 [262].

### 5.1 Broader Relevance of Pre-clinical Models

The corticospinal tract in rodents runs through the dorsal funiculi and ends at the dorsal horn, as opposed to the direct ventral connections in humans [265]. ALS models of non-primates show early corticomotor hyperexcitability, which modulates LMN dysfunction through the local oligosynaptic and excitatory inter-neuronal networks [266]. It is important to use a range of complex disease models, including patient fibroblast-derived neural progenitor differentiation systems, and monitor the same prognostic phenotype marker from disease models to clinical trials.

### 5.2 Stage-specific Non-cell Autonomous Phenotypes

In-depth methodological analysis and holistic integration of epidemiological and clinicopathological data from patient cohorts can help to characterize common and divergent mechanisms in ALS pathophysiology. Modulation of the activity of γ-MNs, Renshaw cells, or metabotropic GluRs with the help of inhibitory ‘designer receptors exclusively activated by designer drugs’ (DREADDs) might be useful in targeting the neuronal microcircuit dysfunction in ALS [61]. Notably, the lack of selectivity and efficacy of the autophagy-inducer drugs tested for ALS therapy makes it necessary to elucidate the site and the stage-specific window of dysfunction [109]. Disaggregases like heat shock proteins HSP27 and HSPB1 can regulate phase separation and liquid-to-gel transition of accumulated TDP43 or stress granule protein G3BP1 condensates in axons and NMJs [267]. The use of 3D microfluidic system models for the stratification of heterogeneous non-neuronal phenotypes may provide new therapeutic targets to combat the non-cell autonomous pathogenicity in ALS [71].

It is sensible to design novel therapies that either target the inhibition of degenerative factors selectively active in ALS-vulnerable motor neurons and myofibres or the activation of intrinsic neuroprotective defence factors expressed in their resilient counterparts [52]. In the early stages of ALS, the synaptic hyperexcitability overactivated BDNF/TrkB signaling, which can be prevented by the inhibition of the adenosine 2A receptor (A2aR) that transactivates TrkB [268]. Development of high-resolution techniques that account for spatial subdivision of metabolic identities across heterogeneous cellular networks is required for the ‘precision medicine’ research in ALS. Machine learning techniques can prove useful in the identification of biomarkers of target engagement as well as disease progression [269].

### 5.3 Stratification of Sporadic Cases

There is a need for integration of the effect of genome, exposome, and behavome on the spatiotemporal patterns of health outcomes across populations. This brings in the concept of genetic geographic information science (Genetic GISc) [270]. Variations in the neuroendocrine stress response can influence the clinical heterogeneity of ALS in a vulnerable genetic, constitutional, or epigenetic background [271].

### 5.4 Role of Hormetic Dose Response in Pathology

An interesting hypothesis of ALS pathogenecity posits that the accumulation of age-associated oxidative damage accounts for the late onset and slow progression of neurodegenerative disorders [272]. Mild exposure to environmental stressors activates the adaptive stress response pathways involving the vitagene network, through a central redox-sensing thiol-modifying signalling, for the synthesis of stress-resistance proteins [273]. This integrated response protects the biological system from severe exposure in the future and constitutes the biphasic ‘preconditioning’ or ‘hormetic’ response [274]. Intriguingly, improvement of prognosis in SOD^1G93A^ mice observed upon exposure to a sub-toxic dose of neurotoxin β-methylamino-L-alanine (L-BMAA) suggests that variation in exposure to an environmental risk factor can be neuroprotective or neurotoxic in the context of a genetic predisposition [275]. Sustained activation of NMDAR leads to prolonged stimulation of NOS, generation of superoxide or peroxynitrite anions, nitric oxide (NO) mediated S-nitrosylation of survival proteins (*e.g.*, MMP9, PDI, COX) and glutamate release-associated synaptic vesicle proteins [272]. However, physiological levels of NO promote survival by nitrosylating NMDAR and Caspase-3 subunits with activation of Akt kinase and CREB signalling [272]. The adaptive nature of hormetic dose responses provides a quantitative index of the plasticity of biological systems irrespective of gender, biological model, timepoint, nature of the agent, or type of induction [274]. To combat both acute and chronic neurodegenerative diseases, the low-dose response stimulation of ER stress and antioxidant pathways can be targeted through dietary restriction or chemically modified drugs [273]. Nutraceuticals such as hydroxytyrosol (Hidrox^®^) can be promising in ALS therapy since it modulates the Nrf2-regulated hemeoxygenase-1 (HO-1) pathway, NF-κB signalling, and sirtuin signalling [276]. Moreover, resveratrol, an activator of SIRT1-FOXO signalling and Keap1-Nrf2 signalling, is known to be neuroprotective in experimental ALS models [277]. The range estimation of stimulatory hormetic dose responses can be used to improve the quantitative design of a clinical study, which increases the probability of inducing an optimal response across the heterogenous patient subgroups [278].

### 5.5 Epigenetic and Epitranscriptomic Precision Research

Novel techniques to detect disease-associated epigenetic marks include methylcytosine-capture DNA hybridization immunoassay for semi-quantitative detection of the repeat methylation levels, especially for the diagnosis of C9orf72 repeat expansion carriers [279]. Notably, a recent study demonstrated direct *in vivo* non-invasive spatial profiling of DNA methylation in mouse brains through a novel 13C tracer-based magnetic resonance spectroscopic imaging (13C-MRSI) method [280]. Regarding ALS epigenetic therapy, research should now be directed toward drugs targeting DNA and histone methylation modifiers and histone phosphorylases. Inhibition of protein phosphatase 1, which erases the H3S10ph mark, can be studied as a potential therapeutic target [281]. Recently, FDA has approved AMX0035 (Relyvrio or Albrizo) for ALS treatment, a combination of taurursodiol and the pan-HDAC inhibitor sodium phenylbutyrate (4-PB), which rescues from ER stress and mitochondrial dysfunction [282]. However, large-scale clinical trials for epigenetic modifier drugs is limited, given the opposing nature of epigenetic modifier activity during ALS and the risk of off-target effects [283].

A bidirectional dCasRx epitranscriptome editing platform composed of a nuclear-localized dCasRx conjugated with methyltransferase or demethylase is currently used to discern the molecular function of individual site-specific m6A modifications or modifiers [284]. RNA metabolism defects have been successfully reversed by CRISPR-Cas9 targeting of repeat expansions in C9orf72 fALS patient cells [285]. Given the cross-talk between epigenetic and epitranscriptomic signatures, it would be prudent to design ‘precision medicine’ regimens for sALS patient subgroups with a defined genetic makeup and exposome.

**Questions for Future Research**

- How do the shared and unique molecular networks of pathogenicity determine a longer survival in ALS cases involving the male gender, spinal form of onset, younger age at onset or diagnosis, and a higher baseline score on the revised ALS Functional Rating Scale (ALSFRS-R) or the Rasch-built Overall ALS Disability Scale (ROADS)?
- How can different environmental factors influence common metabolic pathways to induce changes in the epigenetic or epitranscriptomic landscape in a tissue or cell-specific manner?

Rather than simple association studies, interactions between cellular processes and molecular networks need to be considered for combination therapy or targeting upstream regulators in complex neurodegenerative disorders like ALS.

## CONCLUSION

ALS entails a complex genetic architecture that is oligogenic or polygenic in sporadic cases and mostly monogenic in familial cases. Secondary oligogenic variants with low allele frequency have uncertain or varying effect sizes in the presence of a highly penetrant primary variant, which might result in the pleiotropy of phenotype [286]. Based on various large-scale epidemiological studies, the ALS phenotype has been regarded as the culmination of a six-step process of pathogenesis, with a lesser number of pathogenesis-relevant steps in patient cohorts with large-effect genetic variants (SOD1, C9ORF72) [287]. The disease endophenotype is the end result of the interaction between inherited genetic risk factors and a combination of environmental and endogenous risk factors. The genetic architecture of ALS can be partitioned into distinct risk-associated components rather than simply fALS or sALS, which in turn can facilitate the validation of pathological mechanisms and the stratification of patient sub-groups. The combinatorial effect of interactions between various ALS-linked genetic variants and environmental risk factors can regulate pathogenesis in a synergistic or antagonistic manner [16, 287].

## Figures and Tables

**Fig. (2):**
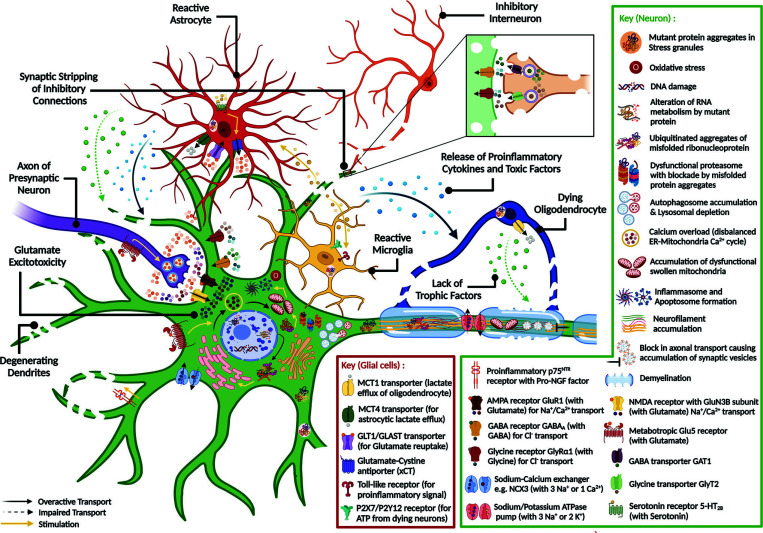
Complex interplay of molecular networks in ALS pathogenesis. Mislocalization and aggregation of misfolded proteins dysregulate RNA metabolism as well as stress granule dynamics, which in turn induces endoplasmic reticulum stress, oxidative stress, mitochondrial dysfunction, and DNA damage. Overexpression of Ca^2+^ -permeable glutamate receptors in postsynaptic neurons, and increased glutamate release by presynaptic neurons and astrocytes is associated with decreased glutamate reuptake from synapse. Concomitant synaptic stripping of the inhibitory connections triggers sustained neuronal firing that results in excitotoxicity. Chemokines released from the affected neurons and reactive astroglia promote necroptosis of oligodendrocytes with subsequent demyelination.

**Fig. (3):**
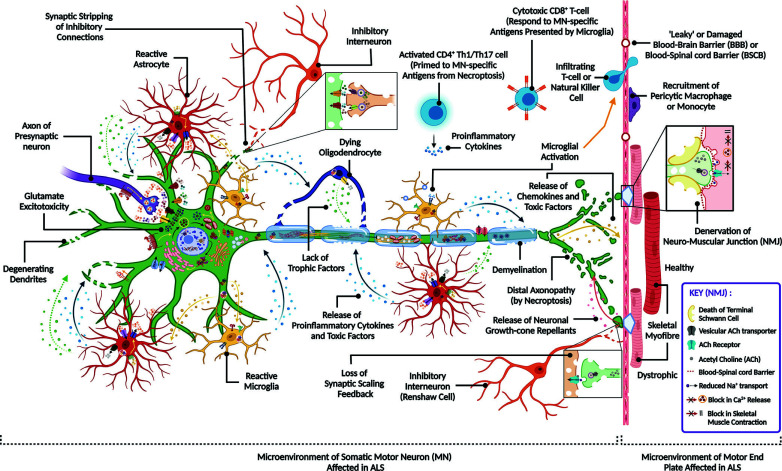
A multi-network and non-cell autonomous failure of cellular homeostasis processes drives ALS. Misfolded protein aggregates induce defects in the intracellular homeostasis processes, which in turn disrupts the protective neuron-astroglia crosstalk. Concomitantly, the defects in synaptic scaling and plasticity of motor neurons results in glutamate excitotoxicity. Release of neuron-specific antigens and proinflammatory stimuli primes T-helper cells into a proinflammatory Th17 phenotype for recruitment into the central nervous system. Side by side, infiltration of cytotoxic T-cells and natural killer cells occurs through adaptive immune signalling. Defective axonal transport hinders the cholinergic transmission towards neuromuscular junctions and impairs myofibre contractility. Release of growth-cone repellents from skeletal muscles and chemorepellents from dying terminal Schwann cells triggers distal axonopathy.

**Figure:**
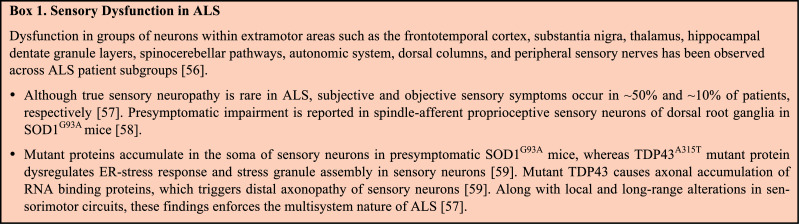


**Figure:**
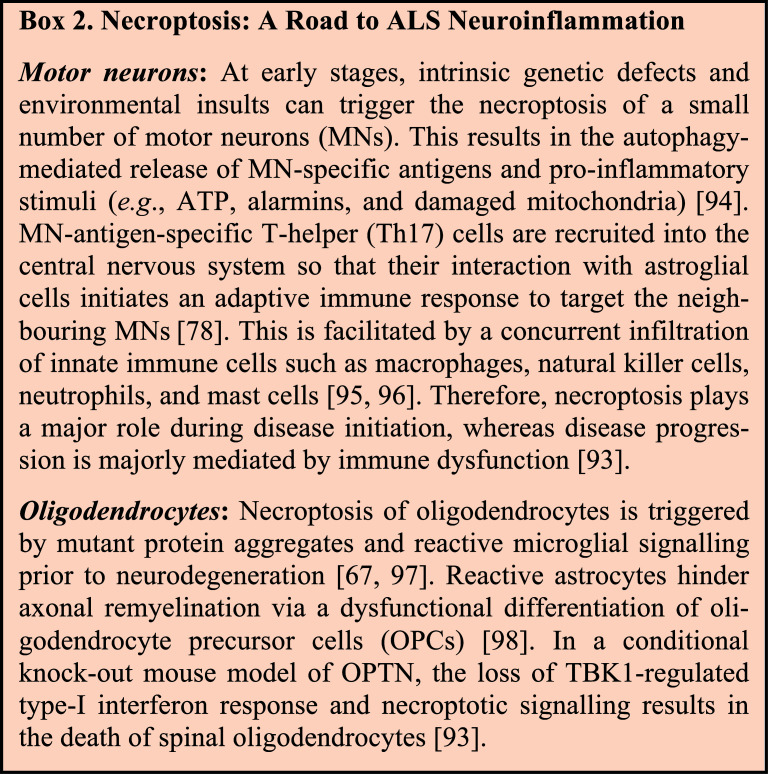


**Figure:**
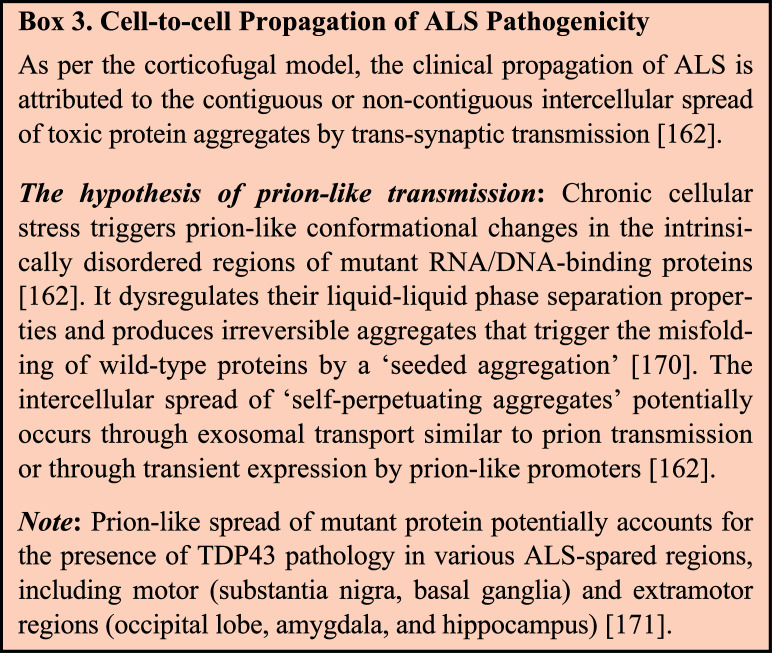


## LIST OF ABBREVIATIONS

ALS: Amyotrophic Lateral Sclerosis
AMPA: α-Amino-3-hydroxy-5-Methyl-4-isoxazole propionic acid
BDNF: Bone Derived Neurotrophic Factor
CSF: Cerebro Spinal Fluid
fALS: Familial ALS
G3BP1: RasGTPase-activating Protein-binding Protein 1
IFN: Interferon
MN: Motor Neuron
mTOR: Mammalian Target of Rapamycin
NF-κB: Nuclear Factor Kappa B
NMDA: N-Methyl-D-Aspartic Acid
NMJ: Neuro Muscular Junction
PGC-1α: Peroxisome Proliferator-activated Receptor Gamma Coactivator 1α
sALS: Sporadic ALS
TIA1: T-cell Intracellular Antigen 1
TNF-α: Tumour Necrosis Factor α

## References

[1] Checkoway H., Lundin J.I., Kelada S.N. (2011). Neurodegenerative diseases.. IARC Sci. Publ..

[2] Logroscino G., Piccininni M., Marin B., Nichols E., Abd-Allah F., Abdelalim A., Alahdab F., Asgedom S.W., Awasthi A., Chaiah Y., Daryani A., Do H.P., Dubey M., Elbaz A., Eskandarieh S., Farhadi F., Farzadfar F., Fereshtehnejad S-M., Fernandes E., Filip I., Foreman K.J., Gebre A.K., Gnedovskaya E.V., Hamidi S., Hay S.I., Irvani S.S.N., Ji J.S., Kasaeian A., Kim Y.J., Mantovani L.G., Mashamba-Thompson T.P., Mehndiratta M.M., Mokdad A.H., Nagel G., Nguyen T.H., Nixon M.R., Olagunju A.T., Owolabi M.O., Piradov M.A., Qorbani M., Radfar A., Reiner R.C., Sahraian M.A., Sarvi S., Sharif M., Temsah O., Tran B.X., Truong N.T., Venketasubramanian N., Winkler A.S., Yimer E.M., Feigin V.L., Vos T., Murray C.J.L. (2018). Global, regional, and national burden of motor neuron diseases 1990–2016: a systematic analysis for the Global Burden of Disease Study 2016.. Lancet Neurol..

[3] Longinetti E., Fang F. (2019). Epidemiology of amyotrophic lateral sclerosis: an update of recent literature.. Curr. Opin. Neurol..

[4] Grad L.I., Rouleau G.A., Ravits J., Cashman N.R. (2017). Clinical spectrum of amyotrophic lateral sclerosis (ALS).. Cold Spring Harb. Perspect. Med..

[5] Abramzon Y.A., Fratta P., Traynor B.J., Chia R. (2020). The overlapping genetics of amyotrophic lateral sclerosis and frontotemporal dementia.. Front. Neurosci..

[6] Masrori P., Van Damme P. (2020). Amyotrophic lateral sclerosis: A clinical review.. Eur. J. Neurol..

[7] Ferraiuolo L., Kirby J., Grierson A.J., Sendtner M., Shaw P.J. (2011). Molecular pathways of motor neuron injury in amyotrophic lateral sclerosis.. Nat. Rev. Neurol..

[8] Manjaly Z.R., Scott K.M., Abhinav K., Wijesekera L., Ganesalingam J., Goldstein L.H., Janssen A., Dougherty A., Willey E., Stanton B.R., Turner M.R., Ampong M.A., Sakel M., Orrell R.W., Howard R., Shaw C.E., Leigh P.N., Al-Chalabi A. (2010). The sex ratio in amyotrophic lateral sclerosis: A population based study.. Amyotroph. Lateral Scler..

[9] Palese F., Sartori A., Verriello L., Ros S., Passadore P., Manganotti P., Barbone F., Pisa F.E. (2019). Epidemiology of amyotrophic lateral sclerosis in Friuli-Venezia Giulia, North-Eastern Italy, 2002–2014: A retrospective population-based study.. Amyotroph. Lateral Scler. Frontotemporal Degener..

[10] Leighton D.J., Newton J., Stephenson L.J., Colville S., Davenport R., Gorrie G., Morrison I., Swingler R., Chandran S., Pal S. (2019). Changing epidemiology of motor neurone disease in Scotland.. J. Neurol..

[11] Chiò A., Logroscino G., Traynor B.J., Collins J., Simeone J.C., Goldstein L.A., White L.A. (2013). Global epidemiology of amyotrophic lateral sclerosis: A systematic review of the published literature.. Neuroepidemiology.

[12] Hardiman O., Al-Chalabi A., Brayne C., Beghi E., van den Berg L.H., Chio A., Martin S., Logroscino G., Rooney J. (2017). The changing picture of amyotrophic lateral sclerosis: Lessons from European registers.. J. Neurol. Neurosurg. Psychiatry.

[13] Brooks B.R., Miller R.G., Swash M., Munsat T.L. (2000). El Escorial revisited: Revised criteria for the diagnosis of amyotrophic lateral sclerosis.. Amyotroph. Lateral Scler. Other Motor Neuron Disord..

[14] van den Berg L.H., Sorenson E., Gronseth G., Macklin E.A., Andrews J., Baloh R.H., Benatar M., Berry J.D., Chio A., Corcia P., Genge A., Gubitz A.K., Lomen-Hoerth C., McDermott C.J., Pioro E.P., Rosenfeld J., Silani V., Turner M.R., Weber M., Brooks B.R., Miller R.G., Mitsumoto H. (2019). Revised Airlie House consensus guidelines for design and implementation of ALS clinical trials.. Neurology.

[15] Shefner J.M., Al-Chalabi A., Baker M.R., Cui L.Y., de Carvalho M., Eisen A., Grosskreutz J., Hardiman O., Henderson R., Matamala J.M., Mitsumoto H., Paulus W., Simon N., Swash M., Talbot K., Turner M.R., Ugawa Y., van den Berg L.H., Verdugo R., Vucic S., Kaji R., Burke D., Kiernan M.C. (2020). A proposal for new diagnostic criteria for ALS.. Clin. Neurophysiol..

[16] Bradley W.G., Andrew A.S., Traynor B.J., Chiò A., Butt T.H., Stommel E.W. (2018). Gene-environment-time interactions in neurodegenerative diseases: Hypotheses and research approaches.. Ann. Neurosci..

[17] Rossi F.H. (2013). Pathophysiology of Amyotrophic Lateral Sclerosis..

[18] Schweingruber C., Hedlund E. (2022). The cell autonomous and non-cell autonomous aspects of neuronal vulnerability and resilience in amyotrophic lateral sclerosis.. Biology.

[19] Turner M.R., Hardiman O., Benatar M., Brooks B.R., Chio A., de Carvalho M., Ince P.G., Lin C., Miller R.G., Mitsumoto H., Nicholson G., Ravits J., Shaw P.J., Swash M., Talbot K., Traynor B.J., Van den Berg L.H., Veldink J.H., Vucic S., Kiernan M.C. (2013). Controversies and priorities in amyotrophic lateral sclerosis.. Lancet Neurol..

[20] Mejzini R., Flynn L.L., Pitout I.L., Fletcher S., Wilton S.D., Akkari P.A. (2019). ALS genetics, mechanisms, and therapeutics: Where are we now?. Front. Neurosci..

[21] Ryan M., Heverin M., McLaughlin R.L., Hardiman O. (2019). Lifetime risk and heritability of amyotrophic lateral sclerosis.. JAMA Neurol..

[22] Fifita J.A., Williams K.L., Sundaramoorthy V., Mccann E.P., Nicholson G.A., Atkin J.D., Blair I.P. (2017). A novel amyotrophic lateral sclerosis mutation in OPTN induces ER stress and Golgi fragmentation *in vitro*.. Amyotroph. Lateral Scler. Frontotemporal Degener..

[23] Brown C. (2017). Non-Familial ALS: A tangled web.. Nature.

[24] Cooper-Knock J., Harvey C., Zhang S., Moll T., Timpanaro I.S., Kenna K.P., Iacoangeli A., Veldink J.H. (2021). Advances in the genetic classification of amyotrophic lateral sclerosis.. Curr. Opin. Neurol..

[25] van Rheenen W., van der Spek R.A.A., Bakker M.K., van Vugt J.J.F.A., Hop P.J., Zwamborn R.A.J., de Klein N., Westra H.J., Bakker O.B., Deelen P., Shireby G., Hannon E., Moisse M., Baird D., Restuadi R., Dolzhenko E., Dekker A.M., Gawor K., Westeneng H.J., Tazelaar G.H.P., van Eijk K.R., Kooyman M., Byrne R.P., Doherty M., Heverin M., Al Khleifat A., Iacoangeli A., Shatunov A., Ticozzi N., Cooper-Knock J., Smith B.N., Gromicho M., Chandran S., Pal S., Morrison K.E., Shaw P.J., Hardy J., Orrell R.W., Sendtner M., Meyer T., Başak N., van der Kooi A.J., Ratti A., Fogh I., Gellera C., Lauria G., Corti S., Cereda C., Sproviero D., D’Alfonso S., Sorarù G., Siciliano G., Filosto M., Padovani A., Chiò A., Calvo A., Moglia C., Brunetti M., Canosa A., Grassano M., Beghi E., Pupillo E., Logroscino G., Nefussy B., Osmanovic A., Nordin A., Lerner Y., Zabari M., Gotkine M., Baloh R.H., Bell S., Vourc’h P., Corcia P., Couratier P., Millecamps S., Meininger V., Salachas F., Mora Pardina J.S., Assialioui A., Rojas-García R., Dion P.A., Ross J.P., Ludolph A.C., Weishaupt J.H., Brenner D., Freischmidt A., Bensimon G., Brice A., Durr A., Payan C.A.M., Saker-Delye S., Wood N.W., Topp S., Rademakers R., Tittmann L., Lieb W., Franke A., Ripke S., Braun A., Kraft J., Whiteman D.C., Olsen C.M., Uitterlinden A.G., Hofman A., Rietschel M., Cichon S., Nöthen M.M., Amouyel P., Comi G., Riva N., Lunetta C., Gerardi F., Cotelli M.S., Rinaldi F., Chiveri L., Guaita M.C., Perrone P., Ceroni M., Diamanti L., Ferrarese C., Tremolizzo L., Delodovici M.L., Bono G., Canosa A., Manera U., Vasta R., Bombaci A., Casale F., Fuda G., Salamone P., Iazzolino B., Peotta L., Cugnasco P., De Marco G., Torrieri M.C., Palumbo F., Gallone S., Barberis M., Sbaiz L., Gentile S., Mauro A., Mazzini L., De Marchi F., Corrado L., D’Alfonso S., Bertolotto A., Gionco M., Leotta D., Odddenino E., Imperiale D., Cavallo R., Pignatta P., De Mattei M., Geda C., Papurello D.M., Gusmaroli G., Comi C., Labate C., Ruiz L., Ferrandi D., Rota E., Aguggia M., Di Vito N., Meineri P., Ghiglione P., Launaro N., Dotta M., Di Sapio A., Giardini G., Tiloca C., Peverelli S., Taroni F., Pensato V., Castellotti B., Comi G.P., Del Bo R., Ceroni M., Gagliardi S., Corrado L., Mazzini L., Raggi F., Simoncini C., Lo Gerfo A., Inghilleri M., Ferlini A., Simone I.L., Passarella B., Guerra V., Zoccolella S., Nozzoli C., Mundi C., Leone M., Zarrelli M., Tamma F., Valluzzi F., Calabrese G., Boero G., Rini A., Traynor B.J., Singleton A.B., Mitne Neto M., Cauchi R.J., Ophoff R.A., Wiedau-Pazos M., Lomen-Hoerth C., van Deerlin V.M., Grosskreutz J., Roediger A., Gaur N., Jörk A., Barthel T., Theele E., Ilse B., Stubendorff B., Witte O.W., Steinbach R., Hübner C.A., Graff C., Brylev L., Fominykh V., Demeshonok V., Ataulina A., Rogelj B., Koritnik B., Zidar J., Ravnik-Glavač M., Glavač D., Stević Z., Drory V., Povedano M., Blair I.P., Kiernan M.C., Benyamin B., Henderson R.D., Furlong S., Mathers S., McCombe P.A., Needham M., Ngo S.T., Nicholson G.A., Pamphlett R., Rowe D.B., Steyn F.J., Williams K.L., Mather K.A., Sachdev P.S., Henders A.K., Wallace L., de Carvalho M., Pinto S., Petri S., Weber M., Rouleau G.A., Silani V., Curtis C.J., Breen G., Glass J.D., Brown R.H., Landers J.E., Shaw C.E., Andersen P.M., Groen E.J.N., van Es M.A., Pasterkamp R.J., Fan D., Garton F.C., McRae A.F., Davey Smith G., Gaunt T.R., Eberle M.A., Mill J., McLaughlin R.L., Hardiman O., Kenna K.P., Wray N.R., Tsai E., Runz H., Franke L., Al-Chalabi A., Van Damme P., van den Berg L.H., Veldink J.H. (2021). Common and rare variant association analyses in amyotrophic lateral sclerosis identify 15 risk loci with distinct genetic architectures and neuron-specific biology.. Nat. Genet..

[26] Casas C., Manzano R., Vaz R., Osta R., Brites D. (2016). Synaptic failure: Focus in an integrative view of ALS.. Brain Plast..

[27] Fogarty M. (2019). Amyotrophic lateral sclerosis as a synaptopathy.. Neural Regen. Res..

[28] Genç B., Jara J.H., Lagrimas A.K.B., Pytel P., Roos R.P., Mesulam M.M., Geula C., Bigio E.H., Özdinler P.H. (2017). Apical dendrite degeneration, a novel cellular pathology for Betz cells in ALS.. Sci. Rep..

[29] Guidotti G., Scarlata C., Brambilla L., Rossi D. (2021). Tumor necrosis factor alpha in amyotrophic lateral sclerosis: Friend or foe?. Cells.

[30] Bursch F., Kalmbach N., Naujock M., Staege S., Eggenschwiler R., Abo-Rady M., Japtok J., Guo W., Hensel N., Reinhardt P., Boeckers T.M., Cantz T., Sterneckert J., Van Den Bosch L., Hermann A., Petri S., Wegner F. (2019). Altered calcium dynamics and glutamate receptor properties in iPSC-derived motor neurons from ALS patients with C9orf72, FUS, SOD1 or TDP43 mutations.. Hum. Mol. Genet..

[31] Bonifacino T., Provenzano F., Gallia E., Ravera S., Torazza C., Bossi S., Ferrando S., Puliti A., Van Den Bosch L., Bonanno G., Milanese M. (2019). *In-vivo* genetic ablation of metabotropic glutamate receptor type 5 slows down disease progression in the SOD^1G93A^ mouse model of amyotrophic lateral sclerosis.. Neurobiol. Dis..

[32] Vermeiren Y., Janssens J., Van Dam D., De Deyn P.P. (2018). Serotonergic dysfunction in amyotrophic lateral sclerosis and parkinson’s disease: Similar mechanisms, dissimilar outcomes.. Front. Neurosci..

[33] Yang Y., Gozen O., Watkins A., Lorenzini I., Lepore A., Gao Y., Vidensky S., Brennan J., Poulsen D., Won Park J., Li Jeon N., Robinson M.B., Rothstein J.D. (2009). Presynaptic regulation of astroglial excitatory neurotransmitter transporter GLT1.. Neuron.

[34] Scamps F., Aimond F., Hilaire C., Raoul C. (2021). Synaptic transmission and motoneuron excitability defects in amyotrophic lateral sclerosis.. Amyotrophic Lateral Sclerosis.

[35] Sunico C.R., Domínguez G., García-Verdugo J.M., Osta R., Montero F., Moreno-López B. (2011). Reduction in the motoneuron inhibitory/excitatory synaptic ratio in an early-symptomatic mouse model of amyotrophic lateral sclerosis.. Brain Pathol..

[36] Sirabella R., Valsecchi V., Anzilotti S., Cuomo O., Vinciguerra A., Cepparulo P., Brancaccio P., Guida N., Blondeau N., Canzoniero L.M.T., Franco C., Amoroso S., Annunziato L., Pignataro G. (2018). Ionic homeostasis maintenance in ALS: Focus on new therapeutic targets.. Front. Neurosci..

[37] Ragagnin A.M.G., Shadfar S., Vidal M., Jamali M.S., Atkin J.D. (2019). Motor neuron susceptibility in ALS/FTD.. Front. Neurosci..

[38] Tateno M., Kato S., Sakurai T., Nukina N., Takahashi R., Araki T. (2009). Mutant SOD1 impairs axonal transport of choline acetyltransferase and acetylcholine release by sequestering KAP3.. Hum. Mol. Genet..

[39] Verma S., Khurana S., Vats A., Sahu B., Ganguly N.K., Chakraborti P., Gourie-Devi M., Taneja V. (2022). Neuromuscular junction dysfunction in amyotrophic lateral sclerosis.. Mol. Neurobiol..

[40] Lin C.Y., Wu C.L., Lee K.Z., Chen Y.J., Zhang P.H., Chang C.Y., Harn H.J., Lin S.Z., Tsai H.J. (2019). Extracellular Pgk1 enhances neurite outgrowth of motoneurons through Nogo66/NgR-independent targeting of NogoA.. eLife.

[41] Venkova K., Christov A., Kamaluddin Z., Kobalka P., Siddiqui S., Hensley K. (2014). Semaphorin 3A signaling through neuropilin-1 is an early trigger for distal axonopathy in the SOD^1G93A^ mouse model of amyotrophic lateral sclerosis.. J. Neuropathol. Exp. Neurol..

[42] Moloney E.B., de Winter F., Verhaagen J. (2014). ALS as a distal axonopathy: Molecular mechanisms affecting neuromuscular junction stability in the presymptomatic stages of the disease.. Front. Neurosci..

[43] Krieger C., Wang S.J.H., Yoo S.H., Harden N. (2016). Adducin at the neuromuscular junction in amyotrophic lateral sclerosis: Hanging on for dear life.. Front. Cell. Neurosci..

[44] Palma E., Reyes-Ruiz J.M., Lopergolo D., Roseti C., Bertollini C., Ruffolo G., Cifelli P., Onesti E., Limatola C., Miledi R., Inghilleri M. (2016). Acetylcholine receptors from human muscle as pharmacological targets for ALS therapy.. Proc. Natl. Acad. Sci. USA.

[45] Van Hoecke A., Schoonaert L., Lemmens R., Timmers M., Staats K.A., Laird A.S., Peeters E., Philips T., Goris A., Dubois B., Andersen P.M., Al-Chalabi A., Thijs V., Turnley A.M., van Vught P.W., Veldink J.H., Hardiman O., Van Den Bosch L., Gonzalez-Perez P., Van Damme P., Brown R.H., van den Berg L.H., Robberecht W. (2012). EPHA4 is a disease modifier of amyotrophic lateral sclerosis in animal models and in humans.. Nat. Med..

[46] Murray L.M., Talbot K., Gillingwater T.H. (2010). Review: Neuromuscular synaptic vulnerability in motor neurone disease: amyotrophic lateral sclerosis and spinal muscular atrophy.. Neuropathol. Appl. Neurobiol..

[47] Schomburg E.D., Steffens H., Zschüntzsch J., Dibaj P., Keller B.U. (2011). Fatigability of spinal reflex transmission in a mouse model (SOD^1G93A^ ) of amyotrophic lateral sclerosis.. Muscle Nerve.

[48] Rocha M.C., Pousinha P.A., Correia A.M., Sebastião A.M., Ribeiro J.A. (2013). Early changes of neuromuscular transmission in the (SOD^1G93A^ ) mice model of ALS start long before motor symptoms onset.. PLoS One.

[49] Carrasco D.I., Seburn K.L., Pinter M.J. (2016). Altered terminal Schwann cell morphology precedes denervation in SOD1 mice.. Exp. Neurol..

[50] Manzano R., Toivonen J.M., Calvo A.C., Oliván S., Zaragoza P., Rodellar C., Montarras D., Osta R. (2013). Altered *in vitro* proliferation of mouse SOD1-G93A skeletal muscle satellite cells.. Neurodegener. Dis..

[51] Nijssen J., Comley L.H., Hedlund E. (2017). Motor neuron vulnerability and resistance in amyotrophic lateral sclerosis.. Acta Neuropathol..

[52] Rochat C., Schneider B.L., Bernard-Marissal N. (2016). Selective vulnerability of neuronal subtypes in ALS: A fertile ground for the identification of therapeutic targets.. Update on Amyotrophic Lateral Sclerosis..

[53] Ruegsegger C., Maharjan N., Goswami A., Filézac de L’Etang A., Weis J., Troost D., Heller M., Gut H., Saxena S. (2016). Aberrant association of misfolded SOD1 with Na^+^/K^+^ATPase-α3 impairs its activity and contributes to motor neuron vulnerability in ALS.. Acta Neuropathol..

[54] Ramírez-Jarquín U.N., Tapia R. (2018). Excitatory and inhibitory neuronal circuits in the spinal cord and their role in the control of motor neuron function and degeneration.. ACS Chem. Neurosci..

[55] Orr B.O., Hauswirth A.G., Celona B., Fetter R.D., Zunino G., Kvon E.Z., Zhu Y., Pennacchio L.A., Black B.L., Davis G.W. (2020). Presynaptic homeostasis opposes disease progression in mouse models of ALS-Like degeneration: Evidence for homeostatic neuroprotection.. Neuron.

[56] Wijesekera L.C., Nigel Leigh P. (2009). Amyotrophic lateral sclerosis.. Orphanet J. Rare Dis..

[57] Isaacs J.D., Dean A.F., Shaw C.E., Al-Chalabi A., Mills K.R., Leigh P.N. (2006). Amyotrophic lateral sclerosis with sensory neuropathy: Part of a multisystem disorder?. J. Neurol. Neurosurg. Psychiatry.

[58] Seki S., Yamamoto T., Quinn K., Spigelman I., Pantazis A., Olcese R., Wiedau-Pazos M., Chandler S.H., Venugopal S. (2019). Circuit-specific early impairment of proprioceptive sensory neurons in the SOD^1G93A^ mouse model for ALS.. J. Neurosci..

[59] Vaughan S.K., Sutherland N.M., Zhang S., Hatzipetros T., Vieira F., Valdez G. (2018). The ALS-inducing factors, TDP43A315T and SOD^1G93A^ , directly affect and sensitize sensory neurons to stress.. Sci. Rep..

[60] Lalancette-Hebert M., Sharma A., Lyashchenko A.K., Shneider N.A. (2016). Gamma motor neurons survive and exacerbate alpha motor neuron degeneration in ALS.. Proc. Natl. Acad. Sci. USA.

[61] Brownstone R.M., Lancelin C. (2018). Escape from homeostasis: spinal microcircuits and progression of amyotrophic lateral sclerosis.. J. Neurophysiol..

[62] Ashford B.A., Boche D., Cooper-Knock J., Heath P.R., Simpson J.E., Highley J.R. (2021). Review: Microglia in motor neuron disease.. Neuropathol. Appl. Neurobiol..

[63] Gomes C., Sequeira C., Barbosa M., Cunha C., Vaz A.R., Brites D. (2020). Astrocyte regional diversity in ALS includes distinct aberrant phenotypes with common and causal pathological processes.. Exp. Cell Res..

[64] Geloso M.C., Corvino V., Marchese E., Serrano A., Michetti F., D’Ambrosi N. (2017). The dual role of microglia in ALS: Mechanisms and therapeutic approaches.. Front. Aging Neurosci..

[65] Trolese M.C., Mariani A., Terao M., de Paola M., Fabbrizio P., Sironi F., Kurosaki M., Bonanno S., Marcuzzo S., Bernasconi P., Trojsi F., Aronica E., Bendotti C., Nardo G. (2020). CXCL13/] CXCR5 signalling is pivotal to preserve motor neurons in amyotrophic lateral sclerosis.. EBioMedicine.

[66] Hensley K., Mhatre M., Mou S., Pye Q.N., Stewart C., West M., Williamson K.S. (2006). On the relation of oxidative stress to neuroinflammation: Lessons learned from the G93A-SOD1 mouse model of amyotrophic lateral sclerosis.. Antioxid. Redox Signal..

[67] Puentes F., Malaspina A., van Noort J.M., Amor S. (2016). Non-neuronal cells in ALS: Role of glial, immune cells and blood-CNS barriers.. Brain Pathol..

[68] Santoni G., Cardinali C., Morelli M., Santoni M., Nabissi M., Amantini C. (2015). Danger- and pathogen-associated molecular patterns recognition by pattern-recognition receptors and ion channels of the transient receptor potential family triggers the inflammasome activation in immune cells and sensory neurons.. J. Neuroinflammation.

[69] Ouali A.N., Schurr C., Olde Heuvel F., Tang L., Li Q., Tasdogan A., Kimbara A., Nettekoven M., Ottaviani G., Raposo C., Röver S., Rogers-Evans M., Rothenhäusler B., Ullmer C., Fingerle J., Grether U., Knuesel I., Boeckers T.M., Ludolph A., Wirth T., Roselli F., Baumann B. (2018). NF‐κB activation in astrocytes drives a stage‐specific beneficial neuroimmunological response in ALS.. EMBO J..

[70] Liddelow S.A., Guttenplan K.A., Clarke L.E., Bennett F.C., Bohlen C.J., Schirmer L., Bennett M.L., Münch A.E., Chung W.S., Peterson T.C., Wilton D.K., Frouin A., Napier B.A., Panicker N., Kumar M., Buckwalter M.S., Rowitch D.H., Dawson V.L., Dawson T.M., Stevens B., Barres B.A. (2017). Neurotoxic reactive astrocytes are induced by activated microglia.. Nature.

[71] Vaz S.H., Pinto S., Sebastião A.M., Brites D., Araki T. (2021). Astrocytes in amyotrophic lateral sclerosis. Amyotroph. Lateral Scler..

[72] Zhao W., Beers D.R., Appel S.H. (2013). Immune-mediated mechanisms in the pathoprogression of amyotrophic lateral sclerosis.. J. Neuroimmune Pharmacol..

[73] Johann S., Heitzer M., Kanagaratnam M., Goswami A., Rizo T., Weis J., Troost D., Beyer C. (2015). NLRP3 inflammasome is expressed by astrocytes in the SOD1 mouse model of ALS and in human sporadic ALS patients.. Glia.

[74] MacLean M., Juranek J., Cuddapah S., López-Díez R., Ruiz H.H., Hu J., Frye L., Li H., Gugger P.F., Schmidt A.M. (2021). Microglia RAGE exacerbates the progression of neurodegeneration within the SOD^1G93A^ murine model of amyotrophic lateral sclerosis in a sex-dependent manner.. J. Neuroinflammation.

[75] Eitan C., Siany A., Barkan E., Olender T., van Eijk K.R., Moisse M., Farhan S.M.K., Danino Y.M., Yanowski E., Marmor-Kollet H., Rivkin N., Yacovzada N.S., Hung S.T., Cooper-Knock J., Yu C.H., Louis C., Masters S.L., Kenna K.P., van der Spek R.A.A., Sproviero W., Al Khleifat A., Iacoangeli A., Shatunov A., Jones A.R., Elbaz-Alon Y., Cohen Y., Chapnik E., Rothschild D., Weissbrod O., Beck G., Ainbinder E., Ben-Dor S., Werneburg S., Schafer D.P., Brown R.H., Shaw P.J., Van Damme P., van den Berg L.H., Phatnani H., Segal E., Ichida J.K., Al-Chalabi A., Veldink J.H., Cooper-Knock J., Kenna K.P., Van Damme P., van den Berg L.H., Hornstein E., Hornstein E. (2022). Whole-genome sequencing reveals that variants in the Interleukin 18 Receptor Accessory Protein 3′UTR protect against ALS.. Nat. Neurosci..

[76] Beers D.R., Henkel J.S., Zhao W., Wang J., Appel S.H. (2008). CD4+ T cells support glial neuroprotection, slow disease progression, and modify glial morphology in an animal model of inherited ALS.. Proc. Natl. Acad. Sci. USA.

[77] Henkel J.S., Beers D.R., Wen S., Rivera A.L., Toennis K.M., Appel J.E., Zhao W., Moore D.H., Powell S.Z., Appel S.H. (2013). Regulatory T‐lymphocytes mediate amyotrophic lateral sclerosis progression and survival.. EMBO Mol. Med..

[78] McCombe P.A., Lee J.D., Woodruff T.M., Henderson R.D. (2020). The peripheral immune system and amyotrophic lateral sclerosis.. Front. Neurol..

[79] Volonté C., Apolloni S., Parisi C., Amadio S. (2016). Purinergic contribution to amyotrophic lateral sclerosis.. Neuropharmacology.

[80] Sta M., Sylva-Steenland R.M.R., Casula M., de Jong J.M.B.V., Troost D., Aronica E., Baas F. (2011). Innate and adaptive immunity in amyotrophic lateral sclerosis: Evidence of complement activation.. Neurobiol. Dis..

[81] Kakaroubas N., Brennan S., Keon M., Saksena N.K. (2019). Pathomechanisms of blood-brain barrier disruption in ALS.. Neurosci. J..

[82] Saul J., Hutchins E., Reiman R., Saul M., Ostrow L.W., Harris B.T., Van Keuren-Jensen K., Bowser R., Bakkar N. (2020). Global alterations to the choroid plexus blood-CSF barrier in amyotrophic lateral sclerosis.. Acta Neuropathol. Commun..

[83] Bowerman M. (2013). The neuroinflammation in the physiopathology of amyotrophic lateral sclerosis.. Curr. Adv. Amyotrophic Lateral Sclerosis.

[84] Jiang L.L., Zhu B., Zhao Y., Li X., Liu T., Pina-Crespo J., Zhou L., Xu W., Rodriguez M.J., Yu H., Cleveland D.W., Ravits J., Da Cruz S., Long T., Zhang D., Huang T.Y., Xu H. (2019). Membralin deficiency dysregulates astrocytic glutamate homeostasis, leading to ALS-like impairment.. J. Clin. Invest..

[85] Yin X., Wang S., Qi Y., Wang X., Jiang H., Wang T., Yang Y., Wang Y., Zhang C., Feng H. (2018). Astrocyte elevated gene-1 is a novel regulator of astrogliosis and excitatory amino acid transporter-2 *via* interplaying with nuclear factor-κB signaling in astrocytes from amyotrophic lateral sclerosis mouse model with hSOD1 G93A mutation.. Mol. Cell. Neurosci..

[86] Rosenblum L.T., Shamamandri-Markandaiah S., Ghosh B., Foran E., Lepore A.C., Pasinelli P., Trotti D. (2017). Mutation of the caspase-3 cleavage site in the astroglial glutamate transporter EAAT2 delays disease progression and extends lifespan in the SOD1-G93A mouse model of ALS.. Exp. Neurol..

[87] Chen L.C., Smith A.P., Ben Y., Zukic B., Ignacio S., Moore D., Lee N.M. (2004). Temporal gene expression patterns in G93A/SOD1 mouse.. Amyotroph. Lateral Scler. Other Motor Neuron Disord..

[88] Lopez-Lopez A., Gamez J., Syriani E., Morales M., Salvado M., Rodríguez M.J., Mahy N., Vidal-Taboada J.M. (2014). CX3CR1 is a modifying gene of survival and progression in amyotrophic lateral sclerosis.. PLoS One.

[89] Tripathi P., Rodriguez-Muela N., Klim J.R., de Boer A.S., Agrawal S., Sandoe J., Lopes C.S., Ogliari K.S., Williams L.A., Shear M., Rubin L.L., Eggan K., Zhou Q. (2017). Reactive astrocytes promote ALS-like degeneration and intracellular protein aggregation in human motor neurons by disrupting autophagy through TGF-β1.. Stem Cell Reports.

[90] Cassina P., Miquel E., Martínez-Palma L., Cassina A. (2021). Glial metabolic reprogramming in amyotrophic lateral sclerosis.. Neuroimmunomodulation.

[91] Moisse K., Strong M.J. (2006). Innate immunity in amyotrophic lateral sclerosis.. Biochim. Biophys. Acta Mol. Basis Dis..

[92] Raffaele S., Boccazzi M., Fumagalli M. (2021). Oligodendrocyte dysfunction in amyotrophic lateral sclerosis: Mechanisms and therapeutic perspectives.. Cells.

[93] Ito Y., Ofengeim D., Najafov A., Das S., Saberi S., Li Y., Hitomi J., Zhu H., Chen H., Mayo L., Geng J., Amin P., DeWitt J.P., Mookhtiar A.K., Florez M., Ouchida A.T., Fan J., Pasparakis M., Kelliher M.A., Ravits J., Yuan J. (2016). RIPK1 mediates axonal degeneration by promoting inflammation and necroptosis in ALS.. Science.

[94] Liu J.F., Zheng O.X., Xin J.G., Chen H.H., Xin J.J. (2017). How are necroptosis, immune dysfunction, and motoneuron death connected in amyotrophic lateral sclerosis?. Neuroimmunol. Neuroinflamm..

[95] Endo F., Komine O., Yamanaka K. (2016). Neuroinflammation in motor neuron disease.. Clin. Exp. Neuroimmunol..

[96] Trias E., King P.H., Si Y., Kwon Y., Varela V., Ibarburu S., Kovacs M., Moura I.C., Beckman J.S., Hermine O., Barbeito L. (2018). Mast cells and neutrophils mediate peripheral motor pathway degeneration in ALS.. JCI Insight.

[97] Kang S.H., Li Y., Fukaya M., Lorenzini I., Cleveland D.W., Ostrow L.W., Rothstein J.D., Bergles D.E. (2013). Degeneration and impaired regeneration of gray matter oligodendrocytes in amyotrophic lateral sclerosis.. Nat. Neurosci..

[98] Filipi T., Hermanova Z., Tureckova J., Vanatko O., Anderova M. (2020). Glial cells—the strategic targets in amyotrophic lateral sclerosis treatment.. J. Clin. Med..

[99] Mishra P.S., Boutej H., Soucy G., Bareil C., Kumar S., Picher-Martel V., Dupré N., Kriz J., Julien J.P. (2020). Transmission of ALS pathogenesis by the cerebrospinal fluid.. Acta Neuropathol. Commun..

[100] Sumitha R., Manjunatha V.M., Sabitha R.K., Alladi P.A., Nalini A., Rao L.T., Chandrasekhar Sagar B.K., Steinbusch H.W.M., Kramer B.W., Sathyaprabha T.N., Raju T.R. (2019). Cerebrospinal fluid from patients with sporadic amyotrophic lateral sclerosis induces degeneration of motor neurons derived from human embryonic stem cells.. Mol. Neurobiol..

[101] Mishra P.S., Vijayalakshmi K., Nalini A., Sathyaprabha T.N., Kramer B.W., Alladi P.A., Raju T.R. (2017). Etiogenic factors present in the cerebrospinal fluid from amyotrophic lateral sclerosis patients induce predominantly pro-inflammatory responses in microglia.. J. Neuroinflammation.

[102] Clement A.M., Nguyen M.D., Roberts E.A., Garcia M.L., Boillée S., Rule M., McMahon A.P., Doucette W., Siwek D., Ferrante R.J., Brown R.H., Julien J.P., Goldstein L.S.B., Cleveland D.W. (2003). Wild-type nonneuronal cells extend survival of SOD1 mutant motor neurons in ALS mice.. Science.

[103] Lobsiger C.S., Boillee S., McAlonis-Downes M., Khan A.M., Feltri M.L., Yamanaka K., Cleveland D.W. (2009). Schwann cells expressing dismutase active mutant SOD1 unexpectedly slow disease progression in ALS mice.. Proc. Natl. Acad. Sci. USA.

[104] Boillée S., Yamanaka K., Lobsiger C.S., Copeland N.G., Jenkins N.A., Kassiotis G., Kollias G., Cleveland D.W. (2006). Onset and progression in inherited ALS determined by motor neurons and microglia.. Science.

[105] Van Harten A.C.M., Phatnani H., Przedborski S. (2021). Non-cell-autonomous pathogenic mechanisms in amyotrophic lateral sclerosis.. Trends Neurosci..

[106] Damme M., Suntio T., Saftig P., Eskelinen E.L. (2015). Autophagy in neuronal cells: general principles and physiological and pathological functions.. Acta Neuropathol..

[107] Sasaki S. (2011). Autophagy in spinal cord motor neurons in sporadic amyotrophic lateral sclerosis.. J. Neuropathol. Exp. Neurol..

[108] Fernando R., Castro J.P., Flore T., Deubel S., Grune T., Ott C. (2020). Age-related maintenance of the autophagy-lysosomal system is dependent on skeletal muscle type.. Oxid. Med. Cell. Longev..

[109] Amin A., Perera N.D., Beart P.M., Turner B.J., Shabanpoor F. (2020). Amyotrophic lateral sclerosis and autophagy: Dysfunction and therapeutic targeting.. Cells.

[110] Fujikake N., Shin M., Shimizu S. (2018). Association between autophagy and neurodegenerative diseases.. Front. Neurosci..

[111] Chen A.I., Xiong L.J., Tong Y.U., Mao M. (2013). Neuroprotective effect of brain-derived neurotrophic factor mediated by autophagy through the PI3K/Akt/mTOR pathway.. Mol. Med. Rep..

[112] Ugolino J., Ji Y.J., Conchina K., Chu J., Nirujogi R.S., Pandey A., Brady N.R., Hamacher-Brady A., Wang J. (2016). Loss of C9orf72 enhances autophagic activity *via* deregulated mTOR and TFEB signaling.. PLoS Genet..

[113] Budini M., Buratti E., Morselli E., Criollo A. (2017). Autophagy and its impact on neurodegenerative diseases: New roles for TDP-43 and C9orf72.. Front. Mol. Neurosci..

[114] Chew J., Cook C., Gendron T.F., Jansen-West K., del Rosso G., Daughrity L.M., Castanedes-Casey M., Kurti A., Stankowski J.N., Disney M.D., Rothstein J.D., Dickson D.W., Fryer J.D., Zhang Y.J., Petrucelli L. (2019). Aberrant deposition of stress granule-resident proteins linked to C9orf72-associated TDP-43 proteinopathy.. Mol. Neurodegener..

[115] Nguyen D.K.H., Thombre R., Wang J. (2019). Autophagy as a common pathway in amyotrophic lateral sclerosis.. Neurosci. Lett..

[116] Oakes J.A., Davies M.C., Collins M.O. (2017). TBK1: a new player in ALS linking autophagy and neuroinflammation.. Mol. Brain.

[117] Tak Y.J., Park J.H., Rhim H., Kang S. (2020). ALS-related mutant SOD1 aggregates interfere with mitophagy by sequestering the autophagy receptor optineurin.. Int. J. Mol. Sci..

[118] Zhang Y.J., Jansen-West K., Xu Y.F., Gendron T.F., Bieniek K.F., Lin W.L., Sasaguri H., Caulfield T., Hubbard J., Daughrity L., Chew J., Belzil V.V., Prudencio M., Stankowski J.N., Castanedes-Casey M., Whitelaw E., Ash P.E.A., DeTure M., Rademakers R., Boylan K.B., Dickson D.W., Petrucelli L. (2014). Aggregation-prone c9FTD/ALS poly(GA) RAN-translated proteins cause neurotoxicity by inducing ER stress.. Acta Neuropathol..

[119] Soo K.Y., Sultana J., King A.E., Atkinson R.A.K., Warraich S.T., Sundaramoorthy V., Blair I., Farg M.A., Atkin J.D. (2015). ALS-associated mutant FUS inhibits macroautophagy which is restored by overexpression of Rab1.. Cell Death Discov..

[120] Purice M.D., Taylor J.P. (2018). Linking hnRNP function to ALS and FTD pathology.. Front. Neurosci..

[121] Renaud L., Picher-Martel V., Codron P., Julien J.P. (2019). Key role of UBQLN2 in pathogenesis of amyotrophic lateral sclerosis and frontotemporal dementia.. Acta Neuropathol. Commun..

[122] Burk K., Pasterkamp R.J. (2019). Disrupted neuronal trafficking in amyotrophic lateral sclerosis.. Acta Neuropathol..

[123] Theunissen F., West P.K., Brennan S., Petrović B., Hooshmand K., Akkari P.A., Keon M., Guennewig B. (2021). New perspectives on cytoskeletal dysregulation and mitochondrial mislocalization in amyotrophic lateral sclerosis.. Transl. Neurodegener..

[124] Kieran D., Hafezparast M., Bohnert S., Dick J.R.T., Martin J., Schiavo G., Fisher E.M.C., Greensmith L. (2005). A mutation in dynein rescues axonal transport defects and extends the life span of ALS mice.. J. Cell Biol..

[125] Shi Y., Lin S., Staats K.A., Li Y., Chang W.H., Hung S.T., Hendricks E., Linares G.R., Wang Y., Son E.Y., Wen X., Kisler K., Wilkinson B., Menendez L., Sugawara T., Woolwine P., Huang M., Cowan M.J., Ge B., Koutsodendris N., Sandor K.P., Komberg J., Vangoor V.R., Senthilkumar K., Hennes V., Seah C., Nelson A.R., Cheng T.Y., Lee S.J.J., August P.R., Chen J.A., Wisniewski N., Hanson-Smith V., Belgard T.G., Zhang A., Coba M., Grunseich C., Ward M.E., van den Berg L.H., Pasterkamp R.J., Trotti D., Zlokovic B.V., Ichida J.K. (2018). Haploinsufficiency leads to neurodegeneration in C9ORF72 ALS/FTD human induced motor neurons.. Nat. Med..

[126] Slowicka K., Vereecke L., van Loo G. (2016). Cellular functions of optineurin in health and disease.. Trends Immunol..

[127] Rademakers R., van Blitterswijk M. (2014). Excess of rare damaging TUBA4A variants suggests cytoskeletal defects in ALS.. Neuron.

[128] Laird F.M., Farah M.H., Ackerley S., Hoke A., Maragakis N., Rothstein J.D., Griffin J., Price D.L., Martin L.J., Wong P.C. (2008). Motor neuron disease occurring in a mutant dynactin mouse model is characterized by defects in vesicular trafficking.. J. Neurosci..

[129] Nicolas A., Kenna K.P., Renton A.E., Ticozzi N., Faghri F., Chia R., Dominov J.A., Kenna B.J., Nalls M.A., Keagle P., Rivera A.M., van Rheenen W., Murphy N.A., van Vugt J.J.F.A., Geiger J.T., Van der Spek R.A., Pliner H.A. (2018). Shankaracharya; Smith, B.N.; Marangi, G.; Topp, S.D.; Abramzon, Y.; Gkazi, A.S.; Eicher, J.D.; Kenna, A.; Mora, G.; Calvo, A.; Mazzini, L.; Riva, N.; Mandrioli, J.; Caponnetto, C.; Battistini, S.; Volanti, P.; La Bella, V.; Conforti, F.L.; Borghero, G.; Messina, S.; Simone, I.L.; Trojsi, F.; Salvi, F.; Logullo, F.O.; D’Alfonso, S.; Corrado, L.; Capasso, M.; Ferrucci, L.; Moreno, C.A.M.; Kamalakaran, S.; Goldstein, D.B.; Gitler, A.D.; Harris, T.; Myers, R.M.; Phatnani, H.; Musunuri, R.L.; Evani, U.S.; Abhyankar, A.; Zody, M.C.; Kaye, J.; Finkbeiner, S.; Wyman, S.K.; LeNail, A.; Lima, L.; Fraenkel, E.; Svendsen, C.N.; Thompson, L.M.; Van Eyk, J.E.; Berry, J.D.; Miller, T.M.; Kolb, S.J.; Cudkowicz, M.; Baxi, E.; Benatar, M.; Taylor, J.P.; Rampersaud, E.; Wu, G.; Wuu, J.; Lauria, G.; Verde, F.; Fogh, I.; Tiloca, C.; Comi, G.P.; Sorarù, G.; Cereda, C.; Corcia, P.; Laaksovirta, H.; Myllykangas, L.; Jansson, L.; Valori, M.; Ealing, J.; Hamdalla, H.; Rollinson, S.; Pickering-Brown, S.; Orrell, R.W.; Sidle, K.C.; Malaspina, A.; Hardy, J.; Singleton, A.B.; Johnson, J.O.; Arepalli, S.; Sapp, P.C.; McKenna-Yasek, D.; Polak, M.; Asress, S.; Al-Sarraj, S.; King, A.; Troakes, C.; Vance, C.; de Belleroche, J.; Baas, F.; ten Asbroek, A.L.M.A.; Muñoz-Blanco, J.L.; Hernandez, D.G.; Ding, J.; Gibbs, J.R.; Scholz, S.W.; Floeter, M.K.; Campbell, R.H.; Landi, F.; Bowser, R.; Pulst, S.M.; Ravits, J.M.; MacGowan, D.J.L.; Kirby, J.; Pioro, E.P.; Pamphlett, R.; Broach, J.; Gerhard, G.; Dunckley, T.L.; Brady, C.B.; Kowall, N.W.; Troncoso, J.C.; Le Ber, I.; Mouzat, K.; Lumbroso, S.; Heiman-Patterson, T.D.; Kamel, F.; Van Den Bosch, L.; Baloh, R.H.; Strom, T.M.; Meitinger, T.; Shatunov, A.; Van Eijk, K.R.; de Carvalho, M.; Kooyman, M.; Middelkoop, B.; Moisse, M.; McLaughlin, R.L.; Van Es, M.A.; Weber, M.; Boylan, K.B.; Van Blitterswijk, M.; Rademakers, R.; Morrison, K.E.; Basak, A.N.; Mora, J.S.; Drory, V.E.; Shaw, P.J.; Turner, M.R.; Talbot, K.; Hardiman, O.; Williams, K.L.; Fifita, J.A.; Nicholson, G.A.; Blair, I.P.; Rouleau, G.A.; Esteban-Pérez, J.; García-Redondo, A.; Al-Chalabi, A.; Rogaeva, E.; Zinman, L.; Ostrow, L.W.; Maragakis, N.J.; Rothstein, J.D.; Simmons, Z.; Cooper-Knock, J.; Brice, A.; Goutman, S.A.; Feldman, E.L.; Gibson, S.B.; Taroni, F.; Ratti, A.; Gellera, C.; Van Damme, P.; Robberecht, W.; Fratta, P.; Sabatelli, M.; Lunetta, C.; Ludolph, A.C.; Andersen, P.M.; Weishaupt, J.H.; Camu, W.; Trojanowski, J.Q.; Van Deerlin, V.M.; Brown, R.H., Jr; van den Berg, L.H.; Veldink, J.H.; Harms, M.B.; Glass, J.D.; Stone, D.J.; Tienari, P.; Silani, V.; Chiò, A.; Shaw, C.E.; Traynor, B.J.; Landers, J.E.; Logullo, F.O.; Simone, I.; Logroscino, G.; Salvi, F.; Bartolomei, I.; Borghero, G.; Murru, M.R.; Costantino, E.; Pani, C.; Puddu, R.; Caredda, C.; Piras, V.; Tranquilli, S.; Cuccu, S.; Corongiu, D.; Melis, M.; Milia, A.; Marrosu, F.; Marrosu, M.G.; Floris, G.; Cannas, A.; Tranquilli, S.; Capasso, M.; Caponnetto, C.; Mancardi, G.; Origone, P.; Mandich, P.; Conforti, F.L.; Cavallaro, S.; Mora, G.; Marinou, K.; Sideri, R.; Penco, S.; Mosca, L.; Lunetta, C.; Pinter, G.L.; Corbo, M.; Riva, N.; Carrera, P.; Volanti, P.; Mandrioli, J.; Fini, N.; Fasano, A.; Tremolizzo, L.; Arosio, A.; Ferrarese, C.; Trojsi, F.; Tedeschi, G.; Monsurrò, M.R.; Piccirillo, G.; Femiano, C.; Ticca, A.; Ortu, E.; La Bella, V.; Spataro, R.; Colletti, T.; Sabatelli, M.; Zollino, M.; Conte, A.; Luigetti, M.; Lattante, S.; Marangi, G.; Santarelli, M.; Petrucci, A.; Pugliatti, M.; Pirisi, A.; Parish, L.D.; Occhineri, P.; Giannini, F.; Battistini, S.; Ricci, C.; Benigni, M.; Cau, T.B.; Loi, D.; Calvo, A.; Moglia, C.; Brunetti, M.; Barberis, M.; Restagno, G.; Casale, F.; Marrali, G.; Fuda, G.; Ossola, I.; Cammarosano, S.; Canosa, A.; Ilardi, A.; Manera, U.; Grassano, M.; Tanel, R.; Pisano, F.; Harms, M.B.; Goldstein, D.B.; Shneider, N.A.; Goutman, S.; Simmons, Z.; Miller, T.M.; Chandran, S.; Pal, S.; Manousakis, G.; Appel, S.H.; Simpson, E.; Wang, L.; Baloh, R.H.; Gibson, S.; Bedlack, R.; Lacomis, D.; Sareen, D.; Sherman, A.; Bruijn, L.; Penny, M.; Allen, A.S.; Appel, S.; Baloh, R.H.; Bedlack, R.S.; Boone, B.E.; Brown, R.; Carulli, J.P.; Chesi, A.; Chung, W.K.; Cirulli, E.T.; Cooper, G.M.; Couthouis, J.; Day-Williams, A.G.; Dion, P.A.; Gibson, S.; Gitler, A.D.; Glass, J.D.; Goldstein, D.B.; Han, Y.; Harms, M.B.; Harris, T.; Hayes, S.D.; Jones, A.L.; Keebler, J.; Krueger, B.J.; Lasseigne, B.N.; Levy, S.E.; Lu, Y-F.; Maniatis, T.; McKenna-Yasek, D.; Miller, T.M.; Myers, R.M.; Petrovski, S.; Pulst, S.M.; Raphael, A.R.; Ravits, J.M.; Ren, Z.; Rouleau, G.A.; Sapp, P.C.; Shneider, N.A.; Simpson, E.; Sims, K.B.; Staropoli, J.F.; Waite, L.L.; Wang, Q.; Wimbish, J.R.; Xin, W.W.; Phatnani, H.; Kwan, J.; Sareen, D.; Broach, J.R.; Simmons, Z.; Arcila-Londono, X.; Lee, E.B.; Van Deerlin, V.M.; Shneider, N.A.; Fraenkel, E.; Ostrow, L.W.; Baas, F.; Zaitlen, N.; Berry, J.D.; Malaspina, A.; Fratta, P.; Cox, G.A.; Thompson, L.M.; Finkbeiner, S.; Dardiotis, E.; Miller, T.M.; Chandran, S.; Pal, S.; Hornstein, E.; MacGowan, D.J.; Heiman-Patterson, T.; Hammell, M.G.; Patsopoulos, N.A.; Dubnau, J.; Nath, A.; Kaye, J.; Finkbeiner, S.; Wyman, S.; LeNail, A.; Lima, L.; Fraenkel, E.; Rothstein, J.D.; Svendsen, C.N.; Thompson, L.M.; Van Eyk, J.; Maragakis, N.J.; Berry, J.D.; Glass, J.D.; Miller, T.M.; Kolb, S.J.; Baloh, R.H.; Cudkowicz, M.; Baxi, E.; Benatar, M.; Taylor, J.P.; Wu, G.; Rampersaud, E.; Wuu, J.; Rademakers, R.; Züchner, S.; Schule, R.; McCauley, J.; Hussain, S.; Cooley, A.; Wallace, M.; Clayman, C.; Barohn, R.; Statland, J.; Ravits, J.; Swenson, A.; Jackson, C.; Trivedi, J.; Khan, S.; Katz, J.; Jenkins, L.; Burns, T.; Gwathmey, K.; Caress, J.; McMillan, C.; Elman, L.; Pioro, E.; Heckmann, J.; So, Y.; Walk, D.; Maiser, S.; Zhang, J.; Silani, V.; Ticozzi, N.; Gellera, C.; Ratti, A.; Taroni, F.; Lauria, G.; Verde, F.; Fogh, I.; Tiloca, C.; Comi, G.P.; Sorarù, G.; Cereda, C.; D’Alfonso, S.; Corrado, L.; De Marchi, F.; Corti, S.; Ceroni, M.; Mazzini, L.; Siciliano, G.; Filosto, M.; Inghilleri, M.; Peverelli, S.; Colombrita, C.; Poletti, B.; Maderna, L.; Del Bo, R.; Gagliardi, S.; Querin, G.; Bertolin, C.; Pensato, V.; Castellotti, B.; Camu, W.; Mouzat, K.; Lumbroso, S.; Corcia, P.; Meininger, V.; Besson, G.; Lagrange, E.; Clavelou, P.; Guy, N.; Couratier, P.; Vourch, P.; Danel, V.; Bernard, E.; Lemasson, G.; Al Kheifat, A.; Al-Chalabi, A.; Andersen, P.; Basak, A.N.; Blair, I.P.; Chio, A.; Cooper-Knock, J.; Corcia, P.; Couratier, P.; de Carvalho, M.; Dekker, A.; Drory, V.; Redondo, A.G.; Gotkine, M.; Hardiman, O.; Hide, W.; Iacoangeli, A.; Glass, J.; Kenna, K.; Kiernan, M.; Kooyman, M.; Landers, J.; McLaughlin, R.; Middelkoop, B.; Mill, J.; Neto, M.M.; Moisse, M.; Pardina, J.M.; Morrison, K.; Newhouse, S.; Pinto, S.; Pulit, S.; Robberecht, W.; Shatunov, A.; Shaw, P.; Shaw, C.; Silani, V.; Sproviero, W.; Tazelaar, G.; Ticozzi, N.; van Damme, P.; van den Berg, L.; van der Spek, R.; van Eijk, K.; van Es, M.; van Rheenen, W.; van Vugt, J.; Veldink, J.; Weber, M.; Williams, K.L.; Zatz, M.; Bauer, D.C.; Twine, N.A. Genome-wide Analyses Identify KIF5A as a Novel ALS Gene.. Neuron.

[130] Ackerley S., Grierson A.J., Banner S., Perkinton M.S., Brownlees J., Byers H.L., Ward M., Thornhill P., Hussain K., Waby J.S., Anderton B.H., Cooper J.D., Dingwall C., Leigh P.N., Shaw C.E., Miller C.C.J. (2004). p38α stress-activated protein kinase phosphorylates neurofilaments and is associated with neurofilament pathology in amyotrophic lateral sclerosis.. Mol. Cell. Neurosci..

[131] Brownlees J., Yates A., Bajaj N.P., Davis D., Anderton B.H., Leigh P.N. (2000). Phosphorylation of neurofilament heavy chain side-arms by stress activated protein kinase-1b/Jun N-terminal kinase-3.. J. Cell Sci..

[132] Deshpande M., Feiger Z., Shilton A.K., Luo C.C., Silverman E., Rodal A.A. (2016). Role of BMP receptor traffic in synaptic growth defects in an ALS model.. Mol. Biol. Cell.

[133] Aoki Y., Manzano R., Lee Y., Dafinca R., Aoki M., Douglas A.G.L., Varela M.A., Sathyaprakash C., Scaber J., Barbagallo P., Vader P., Mäger I., Ezzat K., Turner M.R., Ito N., Gasco S., Ohbayashi N., El Andaloussi S., Takeda S., Fukuda M., Talbot K., Wood M.J.A. (2017). C9orf72 and RAB7L1 regulate vesicle trafficking in amyotrophic lateral sclerosis and frontotemporal dementia.. Brain.

[134] Zhen Y., Stenmark H. (2015). Cellular functions of Rab GTPases at a glance.. J. Cell Sci..

[135] Lai C., Xie C., McCormack S.G., Chiang H.C., Michalak M.K., Lin X., Chandran J., Shim H., Shimoji M., Cookson M.R., Huganir R.L., Rothstein J.D., Price D.L., Wong P.C., Martin L.J., Zhu J.J., Cai H. (2006). Amyotrophic lateral sclerosis 2-deficiency leads to neuronal degeneration in amyotrophic lateral sclerosis through altered AMPA receptor trafficking.. J. Neurosci..

[136] Ritson G.P., Custer S.K., Freibaum B.D., Guinto J.B., Geffel D., Moore J., Tang W., Winton M.J., Neumann M., Trojanowski J.Q., Lee V.M.Y., Forman M.S., Taylor J.P. (2010). TDP-43 mediates degeneration in a novel Drosophila model of disease caused by mutations in VCP/p97.. J. Neurosci..

[137] Gwon Y., Maxwell B.A., Kolaitis R.M., Zhang P., Kim H.J., Taylor J.P. (2021). Ubiquitination of G3BP1 mediates stress granule disassembly in a context-specific manner.. Science.

[138] Bertolin C., Querin G., Bozzoni V., Martinelli I., De Bortoli M., Rampazzo A., Gellera C., Pegoraro E., Sorarù G. (2018). NewFIG 4 gene mutations causing aggressive ALS.. Eur. J. Neurol..

[139] Zhang K., Daigle J.G., Cunningham K.M., Coyne A.N., Ruan K., Grima J.C., Bowen K.E., Wadhwa H., Yang P., Rigo F., Taylor J.P., Gitler A.D., Rothstein J.D., Lloyd T.E. (2018). Stress granule assembly disrupts nucleocytoplasmic transport.. Cell.

[140] Ederle H., Funk C., Abou-Ajram C., Hutten S., Funk E.B.E., Kehlenbach R.H., Bailer S.M., Dormann D. (2018). Nuclear egress of TDP-43 and FUS occurs independently of Exportin-1/CRM1.. Sci. Rep..

[141] Ciryam P., Antalek M., Cid F., Tartaglia G.G., Dobson C.M., Guettsches A.K., Eggers B., Vorgerd M., Marcus K., Kley R.A., Morimoto R.I., Vendruscolo M., Weihl C.C. (2019). A metastable subproteome underlies inclusion formation in muscle proteinopathies.. Acta Neuropathol. Commun..

[142] Yerbury J.J., Farrawell N.E., McAlary L. (2020). Proteome homeostasis dysfunction: A unifying principle in ALS pathogenesis.. Trends Neurosci..

[143] Medinas D.B., Valenzuela V., Hetz C. (2017). Proteostasis disturbance in amyotrophic lateral sclerosis.. Hum. Mol. Genet..

[144] Bendotti C., Marino M., Cheroni C., Fontana E., Crippa V., Poletti A., De Biasi S. (2012). Dysfunction of constitutive and inducible ubiquitin-proteasome system in amyotrophic lateral sclerosis: Implication for protein aggregation and immune response.. Prog. Neurobiol..

[145] Ramesh N., Pandey U.B. (2017). Autophagy dysregulation in ALS: When protein aggregates get out of hand.. Front. Mol. Neurosci..

[146] Prasad A., Bharathi V., Sivalingam V., Girdhar A., Patel B.K. (2019). Molecular mechanisms of TDP-43 misfolding and pathology in amyotrophic lateral sclerosis.. Front. Mol. Neurosci..

[147] Ivanova M.I., Sievers S.A., Guenther E.L., Johnson L.M., Winkler D.D., Galaleldeen A. (2014). Aggregation-triggering segments of SOD1 fibril formation support a common pathway for familial and sporadic ALS.. Proc. Natl. Acad. Sci..

[148] Deng H.X., Zhai H., Bigio E.H., Yan J., Fecto F., Ajroud K., Mishra M., Ajroud-Driss S., Heller S., Sufit R., Siddique N., Mugnaini E., Siddique T. (2010). FUS-immunoreactive inclusions are a common feature in sporadic and non-SOD1 familial amyotrophic lateral sclerosis.. Ann. Neurol..

[149] Pokrishevsky E., Grad L.I., Yousefi M., Wang J., Mackenzie I.R., Cashman N.R. (2012). Aberrant localization of FUS and TDP43 is associated with misfolding of SOD1 in amyotrophic lateral sclerosis.. PLoS One.

[150] Williams K.L., Warraich S.T., Yang S., Solski J.A., Fernando R., Rouleau G.A., Nicholson G.A., Blair I.P. (2012). UBQLN2/ubiquilin 2 mutation and pathology in familial amyotrophic lateral sclerosis.. Neurobiol. Aging.

[151] Schmitz A., Pinheiro M.J., Oertig I., Maharjan N., Saxena S. (2021). Emerging perspectives on dipeptide repeat proteins in C9ORF72 ALS/FTD.. Front. Cell. Neurosci..

[152] Gafson A.R., Barthélemy N.R., Bomont P., Carare R.O., Durham H.D., Julien J.P., Kuhle J., Leppert D., Nixon R.A., Weller R.O., Zetterberg H., Matthews P.M. (2020). Neurofilaments: neurobiological foundations for biomarker applications.. Brain.

[153] Kabashi E., Agar J.N., Strong M.J., Durham H.D. (2012). Impaired proteasome function in sporadic amyotrophic lateral sclerosis.. Amyotroph. Lateral Scler..

[154] Cheroni C., Marino M., Tortarolo M., Veglianese P., De Biasi S., Fontana E., Zuccarello L.V., Maynard C.J., Dantuma N.P., Bendotti C. (2009). Functional alterations of the ubiquitin-proteasome system in motor neurons of a mouse model of familial amyotrophic lateral sclerosis.. Hum. Mol. Genet..

[155] Kabashi E., Agar J.N., Taylor D.M., Minotti S., Durham H.D. (2004). Focal dysfunction of the proteasome: a pathogenic factor in a mouse model of amyotrophic lateral sclerosis.. J. Neurochem..

[156] Kitajima Y., Yoshioka K., Suzuki N. (2020). The ubiquitin–proteasome system in regulation of the skeletal muscle homeostasis and atrophy: from basic science to disorders.. J. Physiol. Sci..

[157] Barthelme D., Jauregui R., Sauer R.T. (2015). An ALS disease mutation in Cdc48/p97 impairs 20S proteasome binding and proteolytic communication.. Protein Sci..

[158] Le N.T.T., Chang L., Kovlyagina I., Georgiou P., Safren N., Braunstein K.E., Kvarta M.D., Van Dyke A.M., LeGates T.A., Philips T., Morrison B.M., Thompson S.M., Puche A.C., Gould T.D., Rothstein J.D., Wong P.C., Monteiro M.J. (2016). Motor neuron disease, TDP-43 pathology, and memory deficits in mice expressing ALS–FTD-linked UBQLN2 mutations.. Proc. Natl. Acad. Sci. USA.

[159] Williams K.L., Topp S., Yang S., Smith B., Fifita J.A., Warraich S.T., Zhang K.Y., Farrawell N., Vance C., Hu X., Chesi A., Leblond C.S., Lee A., Rayner S.L., Sundaramoorthy V., Dobson-Stone C., Molloy M.P., van Blitterswijk M., Dickson D.W., Petersen R.C., Graff-Radford N.R., Boeve B.F., Murray M.E., Pottier C., Don E., Winnick C., McCann E.P., Hogan A., Daoud H., Levert A., Dion P.A., Mitsui J., Ishiura H., Takahashi Y., Goto J., Kost J., Gellera C., Gkazi A.S., Miller J., Stockton J., Brooks W.S., Boundy K., Polak M., Muñoz-Blanco J.L., Esteban-Pérez J., Rábano A., Hardiman O., Morrison K.E., Ticozzi N., Silani V., de Belleroche J., Glass J.D., Kwok J.B.J., Guillemin G.J., Chung R.S., Tsuji S., Brown R.H., García-Redondo A., Rademakers R., Landers J.E., Gitler A.D., Rouleau G.A., Cole N.J., Yerbury J.J., Atkin J.D., Shaw C.E., Nicholson G.A., Blair I.P. (2016). CCNF mutations in amyotrophic lateral sclerosis and frontotemporal dementia.. Nat. Commun..

[160] Ling S.C., Polymenidou M., Cleveland D.W. (2013). Converging mechanisms in ALS and FTD: disrupted RNA and protein homeostasis.. Neuron.

[161] Dudman J., Qi X. (2020). Stress Granule Dysregulation in Amyotrophic Lateral Sclerosis.. Front. Cell. Neurosci..

[162] McAlary L., Plotkin S.S., Yerbury J.J., Cashman N.R. (2019). Prion-like propagation of protein misfolding and aggregation in amyotrophic lateral sclerosis.. Front. Mol. Neurosci..

[163] Nolan M., Talbot K., Ansorge O. (2016). Pathogenesis of FUS-associated ALS and FTD: insights from rodent models.. Acta Neuropathol. Commun..

[164] Kitamura A., Nakayama Y., Shibasaki A., Taki A., Yuno S., Takeda K., Yahara M., Tanabe N., Kinjo M. (2016). Interaction of RNA with a C-terminal fragment of the amyotrophic lateral sclerosis-associated TDP43 reduces cytotoxicity.. Sci. Rep..

[165] Birsa N., Bentham M.P., Fratta P. (2020). Cytoplasmic functions of TDP-43 and FUS and their role in ALS.. Semin. Cell Dev. Biol..

[166] Ratti A., Buratti E. (2016). Physiological functions and pathobiology of TDP-43 and FUS/TLS proteins.. J. Neurochem..

[167] Balendra R., Isaacs A.M. (2018). C9orf72-mediated ALS and FTD: multiple pathways to disease.. Nat. Rev. Neurol..

[168] Tang X., Toro A. (2020). T G, S.; Gao, J.; Chalk, J.; Oskarsson, B.; Zhang, K. Divergence, convergence, and therapeutic implications: A cell biology perspective of C9ORF72-ALS/FTD.. Mol. Neurodegener..

[169] Ayaki T., Ito H., Komure O., Kamada M., Nakamura M., Wate R., Kusaka H., Yamaguchi Y., Li F., Kawakami H., Urushitani M., Takahashi R. (2018). Multiple proteinopathies in familial ALS cases with optineurin mutations.. J. Neuropathol. Exp. Neurol..

[170] Münch C., O’Brien J. (2011). Bertolotti, A Prion-like propagation of mutant superoxide dismutase-1 misfolding in neuronal cells.. Proc. Natl. Acad. Sci..

[171] Geser F., Brandmeir N.J., Kwong L.K., Martinez-Lage M., Elman L., McCluskey L., Xie S.X., Lee V.M.Y., Trojanowski J.Q. (2008). Evidence of multisystem disorder in whole-brain map of pathological TDP-43 in amyotrophic lateral sclerosis.. Arch. Neurol..

[172] Sun Y., Curle A.J., Haider A.M., Balmus G. (2020). The role of DNA damage response in amyotrophic lateral sclerosis.. Essays Biochem..

[173] Hewitt G., Carroll B., Sarallah R., Correia-Melo C., Ogrodnik M., Nelson G., Otten E.G., Manni D., Antrobus R., Morgan B.A., von Zglinicki T., Jurk D., Seluanov A., Gorbunova V., Johansen T., Passos J.F., Korolchuk V.I. (2016). SQSTM1/p62 mediates crosstalk between autophagy and the UPS in DNA repair.. Autophagy.

[174] Konopka A., Whelan D.R., Jamali M.S., Perri E., Shahheydari H., Toth R.P., Parakh S., Robinson T., Cheong A., Mehta P., Vidal M., Ragagnin A.M.G., Khizhnyak I., Jagaraj C.J., Galper J., Grima N., Deva A., Shadfar S., Nicholson G.A., Yang S., Cutts S.M., Horejsi Z., Bell T.D.M., Walker A.K., Blair I.P., Atkin J.D. (2020). Impaired NHEJ repair in amyotrophic lateral sclerosis is associated with TDP-43 mutations.. Mol. Neurodegener..

[175] Wang H., Guo W., Mitra J., Hegde P.M., Vandoorne T., Eckelmann B.J., Mitra S., Tomkinson A.E., Van Den Bosch L., Hegde M.L. (2018). Mutant FUS causes DNA ligation defects to inhibit oxidative damage repair in Amyotrophic Lateral Sclerosis.. Nat. Commun..

[176] Kawaguchi T., Rollins M.G., Moinpour M., Morera A.A., Ebmeier C.C., Old W.M., Schwartz J.C. (2020). Changes to the TDP-43 and FUS Interactomes Induced by DNA Damage.. J. Proteome Res..

[177] Haeusler A.R., Donnelly C.J., Periz G., Simko E.A.J., Shaw P.G., Kim M.S., Maragakis N.J., Troncoso J.C., Pandey A., Sattler R., Rothstein J.D., Wang J. (2014). C9orf72 nucleotide repeat structures initiate molecular cascades of disease.. Nature.

[178] Farg M.A., Konopka A., Soo K.Y., Ito D., Atkin J.D. (2017). The DNA damage response (DDR) is induced by the C9orf72 repeat expansion in amyotrophic lateral sclerosis.. Hum. Mol. Genet..

[179] Nihei Y., Mori K., Werner G., Arzberger T., Zhou Q., Khosravi B., Japtok J., Hermann A., Sommacal A., Weber M., Kamp F., Nuscher B., Edbauer D., Haass C. (2020). Poly-glycine–alanine exacerbates C9orf72 repeat expansion-mediated DNA damage *via* sequestration of phosphorylated ATM and loss of nuclear hnRNPA3.. Acta Neuropathol..

[180] Kok J.R., Palminha N.M., Dos Santos Souza C., El-Khamisy S.F., Ferraiuolo L. (2021). DNA damage as a mechanism of neurodegeneration in ALS and a contributor to astrocyte toxicity.. Cell. Mol. Life Sci..

[181] Zhang Y.J., Guo L., Gonzales P.K., Gendron T.F., Wu Y., Jansen-West K., O’Raw A.D., Pickles S.R., Prudencio M., Carlomagno Y., Gachechiladze M.A., Ludwig C., Tian R., Chew J., DeTure M., Lin W.L., Tong J., Daughrity L.M., Yue M., Song Y., Andersen J.W., Castanedes-Casey M., Kurti A., Datta A., Antognetti G., McCampbell A., Rademakers R., Oskarsson B., Dickson D.W., Kampmann M., Ward M.E., Fryer J.D., Link C.D., Shorter J., Petrucelli L. (2019). Heterochromatin anomalies and double-stranded RNA accumulation underlie C9orf72 poly(PR) toxicity.. Science.

[182] Tanaka Y., Chen Z.J. (2012). STING specifies IRF3 phosphorylation by TBK1 in the cytosolic DNA signaling pathway.. Sci. Signal..

[183] Tadic V., Prell T., Lautenschlaeger J., Grosskreutz J. (2014). The ER mitochondria calcium cycle and ER stress response as therapeutic targets in amyotrophic lateral sclerosis.. Front. Cell. Neurosci..

[184] Stoica R., Paillusson S., Gomez-Suaga P., Mitchell J.C., Lau D.H.W., Gray E.H., Sancho R.M., Vizcay-Barrena G., De Vos K.J., Shaw C.E., Hanger D.P., Noble W., Miller C.C.J. (2016). ALS/FTD ‐associated FUS activates GSK ‐3β to disrupt the VAPB – PTPIP 51 interaction and ER –mitochondria associations.. EMBO Rep..

[185] Vicencio E., Beltrán S., Labrador L., Manque P., Nassif M., Woehlbier U. (2020). Implications of selective autophagy dysfunction for ALS pathology.. Cells.

[186] Sprenkle N.T., Sims S.G., Sánchez C.L., Meares G.P. (2017). Endoplasmic reticulum stress and inflammation in the central nervous system.. Mol. Neurodegener..

[187] Lee D.Y., Jeon G.S., Sung J.J. (2020). ALS-Linked Mutant SOD1 associates with TIA-1 and alters stress granule dynamics.. Neurochem. Res..

[188] Matus S., Valenzuela V., Medinas D.B., Hetz C. (2013). Er dysfunction and protein folding stress in ALS.. Int. J. Cell Biol..

[189] Perri E., Parakh S., Atkin J. (2017). Protein Disulphide Isomerases: emerging roles of PDI and ERp57 in the nervous system and as therapeutic targets for ALS.. Expert Opin. Ther. Targets.

[190] Wang L., Popko B., Roos R.P. (2011). The unfolded protein response in familial amyotrophic lateral sclerosis.. Hum. Mol. Genet..

[191] Borgese N., Iacomino N., Colombo S.F., Navone F. (2021). The Link between VAPB loss of function and amyotrophic lateral sclerosis.. Cells.

[192] Sundaramoorthy V., Sultana J.M., Atkin J.D. (2015). Golgi fragmentation in amyotrophic lateral sclerosis, an overview of possible triggers and consequences.. Front. Neurosci..

[193] van Dis V., Kuijpers M., Haasdijk E.D., Teuling E., Oakes S.A., Hoogenraad C.C., Jaarsma D. (2014). Golgi fragmentation precedes neuromuscular denervation and is associated with endosome abnormalities in SOD1-ALS mouse motor neurons.. Acta Neuropathol. Commun..

[194] Sasaki S., Iwata M. (2007). Mitochondrial alterations in the spinal cord of patients with sporadic amyotrophic lateral sclerosis.. J. Neuropathol. Exp. Neurol..

[195] Singh T., Jiao Y., Ferrando L.M., Yablonska S., Li F., Horoszko E.C., Lacomis D., Friedlander R.M., Carlisle D.L. (2021). Neuronal mitochondrial dysfunction in sporadic amyotrophic lateral sclerosis is developmentally regulated.. Sci. Rep..

[196] Thau N., Knippenberg S., Körner S., Rath K.J., Dengler R., Petri S. (2012). Decreased mRNA expression of PGC-1α and PGC-1α-regulated factors in the SOD^1G93A^ ALS mouse model and in human sporadic ALS.. J. Neuropathol. Exp. Neurol..

[197] Moller A., Bauer C.S., Cohen R.N., Webster C.P., De Vos K.J. (2017). Amyotrophic lateral sclerosis-associated mutant SOD1 inhibits anterograde axonal transport of mitochondria by reducing Miro1 levels.. Hum. Mol. Genet..

[198] Davis S.A., Itaman S., Khalid-Janney C.M., Sherard J.A., Dowell J.A., Cairns N.J., Gitcho M.A. (2018). TDP-43 interacts with mitochondrial proteins critical for mitophagy and mitochondrial dynamics.. Neurosci. Lett..

[199] Chen J., Bassot A., Giuliani F., Simmen T. (2021). Amyotrophic lateral sclerosis (ALS): Stressed by dysfunctional mitochondria-endoplasmic reticulum contacts (MERCs).. Cells.

[200] Wang T., Liu H., Itoh K., Oh S., Zhao L., Murata D., Sesaki H., Hartung T., Na C.H., Wang J. (2021). C9orf72 regulates energy homeostasis by stabilizing mitochondrial complex I assembly.. Cell Metab..

[201] Obrador E., Salvador R., López-Blanch R., Jihad-Jebbar A., Vallés S.L., Estrela J.M. (2020). Oxidative stress, neuroinflammation and mitochondria in the pathophysiology of amyotrophic lateral sclerosis.. Antioxidants.

[202] Smith E.F., Shaw P.J., De Vos K.J. (2019). The role of mitochondria in amyotrophic lateral sclerosis.. Neurosci. Lett..

[203] Kazama M., Kato Y., Kakita A., Noguchi N., Urano Y., Masui K., Niida-Kawaguchi M., Yamamoto T., Watabe K., Kitagawa K., Shibata N. (2020). Astrocytes release glutamate *via* cystine/glutamate antiporter upregulated in response to increased oxidative stress related to sporadic amyotrophic lateral sclerosis.. Neuropathology.

[204] Pollari E., Goldsteins G., Bart G., Koistinaho J., Giniatullin R. (2014). The role of oxidative stress in degeneration of the neuromuscular junction in amyotrophic lateral sclerosis.. Front. Cell. Neurosci..

[205] Tsang C.K., Liu Y., Thomas J., Zhang Y., Zheng X.F.S. (2014). Superoxide dismutase 1 acts as a nuclear transcription factor to regulate oxidative stress resistance.. Nat. Commun..

[206] Goh C.W., Lee I.C., Sundaram J.R., George S.E., Yusoff P., Brush M.H., Sze N.S.K., Shenolikar S. (2018). Chronic oxidative stress promotes GADD34-mediated phosphorylation of the TAR DNA-binding protein TDP-43, a modification linked to neurodegeneration.. J. Biol. Chem..

[207] Jagaraj C.J., Parakh S., Atkin J.D. (2021). Emerging evidence highlighting the importance of redox dysregulation in the pathogenesis of amyotrophic lateral sclerosis (ALS).. Front. Cell. Neurosci..

[208] Zala D., Hinckelmann M.V., Yu H., Lyra da Cunha M.M., Liot G., Cordelières F.P., Marco S., Saudou F. (2013). Vesicular glycolysis provides on-board energy for fast axonal transport.. Cell.

[209] Wang T., Tian X., Kim H.B., Jang Y., Huang Z., Na C.H., Wang J. (2022). Intracellular energy controls dynamics of stress-induced ribonucleoprotein granules.. Nat. Commun..

[210] Rodriguez-Rodriguez P., Fernandez E., Almeida A., Bolaños J.P. (2012). Excitotoxic stimulus stabilizes PFKFB3 causing pentose-phosphate pathway to glycolysis switch and neurodegeneration.. Cell Death Differ..

[211] Vandoorne T., De Bock K., Van Den Bosch L. (2018). Energy metabolism in ALS: an underappreciated opportunity?. Acta Neuropathol..

[212] Pennetta G., Welte M.A. (2018). Emerging links between lipid droplets and motor neuron diseases.. Dev. Cell.

[213] Cistaro A., Pagani M., Montuschi A., Calvo A., Moglia C., Canosa A., Restagno G., Brunetti M., Traynor B.J., Nobili F., Carrara G., Fania P., Lopiano L., Valentini M.C., Chiò A. (2014). The metabolic signature of C9ORF72-related ALS: FDG PET comparison with nonmutated patients.. Eur. J. Nucl. Med. Mol. Imaging.

[214] Marini C., Morbelli S., Cistaro A., Campi C., Caponnetto C., Bauckneht M., Bellini A., Buschiazzo A., Calamia I., Beltrametti M.C., Margotti S., Fania P., Poggi I., Cabona C., Capitanio S., Piva R., Calvo A., Moglia C., Canosa A., Massone A., Nobili F., Mancardi G., Chiò A., Piana M., Sambuceti G. (2018). Interplay between spinal cord and cerebral cortex metabolism in amyotrophic lateral sclerosis.. Brain.

[215] Bauckneht M., Lai R., Miceli A., Schenone D., Cossu V., Donegani M.I., Raffa S., Borra A., Marra S., Campi C., Orengo A., Massone A.M., Tagliafico A., Caponnetto C., Cabona C., Cistaro A., Chiò A., Morbelli S., Nobili F., Sambuceti G., Piana M., Marini C. (2020). Spinal cord hypermetabolism extends to skeletal muscle in amyotrophic lateral sclerosis: a computational approach to [18F]-fluorodeoxyglucose PET/CT images.. EJNMMI Res..

[216] Miyazaki K., Masamoto K., Morimoto N., Kurata T., Mimoto T., Obata T., Kanno I., Abe K. (2012). Early and progressive impairment of spinal blood flow-glucose metabolism coupling in motor neuron degeneration of ALS model mice.. J. Cereb. Blood Flow Metab..

[217] Dodge J.C., Treleaven C.M., Fidler J.A., Tamsett T.J., Bao C., Searles M., Taksir T.V., Misra K., Sidman R.L., Cheng S.H., Shihabuddin L.S. (2013). Metabolic signatures of amyotrophic lateral sclerosis reveal insights into disease pathogenesis.. Proc. Natl. Acad. Sci. USA.

[218] Tefera T.W., Steyn F.J., Ngo S.T., Borges K. (2021). CNS glucose metabolism in Amyotrophic Lateral Sclerosis: a therapeutic target?. Cell Biosci..

[219] Steyn F.J., Li R., Kirk S.E., Tefera T.W., Xie T.Y., Tracey T.J., Kelk D., Wimberger E., Garton F.C., Roberts L., Chapman S.E., Coombes J.S., Leevy W.M., Ferri A., Valle C., René F., Loeffler J.P., McCombe P.A., Henderson R.D., Ngo S.T. (2020). Altered skeletal muscle glucose-fatty acid flux in amyotrophic lateral sclerosis.. Brain Commun..

[220] Tefera T.W., Borges K. (2019). Neuronal glucose metabolism is impaired while astrocytic TCA cycling is unaffected at symptomatic stages in the hSOD1 G93A mouse model of amyotrophic lateral sclerosis.. J. Cereb. Blood Flow Metab..

[221] Lee H., Lee J.J., Park N.Y., Dubey S.K., Kim T., Ruan K., Lim S.B., Park S.H., Ha S., Kovlyagina I., Kim K., Kim S., Oh Y., Kim H., Kang S.U., Song M.R., Lloyd T.E., Maragakis N.J., Hong Y.B., Eoh H., Lee G. (2021). Multi-omic analysis of selectively vulnerable motor neuron subtypes implicates altered lipid metabolism in ALS.. Nat. Neurosci..

[222] Palamiuc L., Schlagowski A., Ngo S.T., Vernay A., Dirrig-Grosch S., Henriques A., Boutillier A.L., Zoll J., Echaniz-Laguna A., Loeffler J.P., René F. (2015). A metabolic switch toward lipid use in glycolytic muscle is an early pathologic event in a mouse model of amyotrophic lateral sclerosis.. EMBO Mol. Med..

[223] Yudkoff M., Daikhin Y., Horyn O., Nissim I., Nissim I. (2008). Ketosis and brain handling of glutamate, glutamine, and GABA.. Epilepsia.

[224] Scaricamazza S., Salvatori I., Giacovazzo G., Loeffler J.P., Renè F., Rosina M., Quessada C., Proietti D., Heil C., Rossi S., Battistini S., Giannini F., Volpi N., Steyn F.J., Ngo S.T., Ferraro E., Madaro L., Coccurello R., Valle C., Ferri A. (2020). Skeletal-muscle metabolic reprogramming in ALS-SOD^1G93A^ mice predates disease onset and is a promising therapeutic target.. iScience.

[225] Szelechowski M., Amoedo N., Obre E., Léger C., Allard L., Bonneu M., Claverol S., Lacombe D., Oliet S., Chevallier S., Le Masson G., Rossignol R. (2018). Metabolic reprogramming in amyotrophic lateral sclerosis.. Sci. Rep..

[226] Dodge J.C., Jensen E.H., Yu J., Sardi S.P., Bialas A.R., Taksir T.V., Bangari D.S., Shihabuddin L.S. (2020). Neutral lipid cacostasis contributes to disease pathogenesis in amyotrophic lateral sclerosis.. J. Neurosci..

[227] Henriques A., Huebecker M., Blasco H., Keime C., Andres C.R., Corcia P., Priestman D.A., Platt F.M., Spedding M., Loeffler J.P. (2017). Inhibition of β-Glucocerebrosidase activity preserves motor unit integrity in a mouse model of amyotrophic lateral sclerosis.. Sci. Rep..

[228] Sipione S., Monyror J., Galleguillos D., Steinberg N., Kadam V. (2020). Gangliosides in the brain: Physiology, pathophysiology and therapeutic applications.. Front. Neurosci..

[229] Tracey T.J., Steyn F.J., Wolvetang E.J., Ngo S.T. (2018). Neuronal lipid metabolism: multiple pathways driving functional outcomes in health and disease.. Front. Mol. Neurosci..

[230] Schmitt F., Hussain G., Dupuis L., Loeffler J.P., Henriques A. (2014). A plural role for lipids in motor neuron diseases: Energy, signaling and structure.. Front. Cell. Neurosci..

[231] Mouzat K., Molinari N., Kantar J., Polge A., Corcia P., Couratier P., Clavelou P., Juntas-Morales R., Pageot N., Lobaccaro J.M.A., Raoul C., Lumbroso S., Camu W. (2018). Liver X receptor genes variants modulate ALS phenotype.. Mol. Neurobiol..

[232] Wills A.M., Hubbard J., Macklin E.A., Glass J., Tandan R., Simpson E.P., Brooks B., Gelinas D., Mitsumoto H., Mozaffar T., Hanes G.P., Ladha S.S., Heiman-Patterson T., Katz J., Lou J.S., Mahoney K., Grasso D., Lawson R., Yu H., Cudkowicz M. (2014). Hypercaloric enteral nutrition in patients with amyotrophic lateral sclerosis: a randomised, double-blind, placebo-controlled phase 2 trial.. Lancet.

[233] Fang F., Ingre C., Roos P., Kamel F., Piehl F. (2015). Risk factors for amyotrophic lateral sclerosis.. Clin. Epidemiol..

[234] Goutman S.A., Feldman E.L. (2020). Voicing the Need for Amyotrophic Lateral Sclerosis Environmental Research.. JAMA Neurol..

[235] Allis C.D., Jenuwein T. (2016). The molecular hallmarks of epigenetic control.. Nat. Rev. Genet..

[236] Worpenberg L., Paolantoni C., Roignant J-Y. (2021). Functional interplay within the epitranscriptome: Reality or fiction?. BioEssays.

[237] Appleby-Mallinder C., Schaber E., Kirby J., Shaw P.J., Cooper-Knock J., Heath P.R., Highley J.R. (2021). TDP43 proteinopathy is associated with aberrant DNA methylation in human amyotrophic lateral sclerosis.. Neuropathol. Appl. Neurobiol..

[238] Ozyurt T., Gautam M. (2021). Differential epigenetic signature of corticospinal motor neurons in ALS.. Brain Sci..

[239] Xi Z., Zhang M., Bruni A.C., Maletta R.G., Colao R., Fratta P., Polke J.M., Sweeney M.G., Mudanohwo E., Nacmias B., Sorbi S., Tartaglia M.C., Rainero I., Rubino E., Pinessi L., Galimberti D., Surace E.I., McGoldrick P., McKeever P., Moreno D., Sato C., Liang Y., Keith J., Zinman L., Robertson J., Rogaeva E. (2015). The C9orf72 repeat expansion itself is methylated in ALS and FTLD patients.. Acta Neuropathol..

[240] Wong M., Gertz B., Chestnut B.A., Martin L.J. (2013). Mitochondrial DNMT3A and DNA methylation in skeletal muscle and CNS of transgenic mouse models of ALS.. Front. Cell. Neurosci..

[241] Simpson C.L., Lemmens R., Miskiewicz K., Broom W.J., Hansen V.K., van Vught P.W.J., Landers J.E., Sapp P., Van Den Bosch L., Knight J., Neale B.M., Turner M.R., Veldink J.H., Ophoff R.A., Tripathi V.B., Beleza A., Shah M.N., Proitsi P., Van Hoecke A., Carmeliet P., Horvitz H.R., Leigh P.N., Shaw C.E., van den Berg L.H., Sham P.C., Powell J.F., Verstreken P., Brown R.H., Robberecht W., Al-Chalabi A. (2009). Variants of the elongator protein 3 (ELP3) gene are associated with motor neuron degeneration.. Hum. Mol. Genet..

[242] Taes I., Timmers M., Hersmus N., Bento-Abreu A., Van Den Bosch L., Van Damme P., Auwerx J., Robberecht W. (2013). Hdac6 deletion delays disease progression in the SOD^1G93A^ mouse model of ALS.. Hum. Mol. Genet..

[243] Chen S., Zhang X.J., Li L.X., Wang Y., Zhong R.J., Le W. (2015). Histone deacetylase 6 delays motor neuron degeneration by ameliorating the autophagic flux defect in a transgenic mouse model of amyotrophic lateral sclerosis.. Neurosci. Bull..

[244] Pigna E., Simonazzi E., Sanna K., Bernadzki K.M., Proszynski T., Heil C., Palacios D., Adamo S., Moresi V. (2019). Histone deacetylase 4 protects from denervation and skeletal muscle atrophy in a murine model of amyotrophic lateral sclerosis.. EBioMedicine.

[245] Tibshirani M., Tradewell M.L., Mattina K.R., Minotti S., Yang W., Zhou H., Strong M.J., Hayward L.J., Durham H.D. (2015). Cytoplasmic sequestration of FUS/TLS associated with ALS alters histone marks through loss of nuclear protein arginine methyltransferase 1.. Hum. Mol. Genet..

[246] Masala A., Sanna S., Esposito S., Rassu M., Galioto M., Zinellu A., Carru C., Carrì M.T., Iaccarino C., Crosio C. (2018). Epigenetic changes associated with the expression of amyotrophic lateral sclerosis (ALS) causing genes.. Neuroscience.

[247] Vijayakumar U.G., Milla V., Cynthia Stafford M.Y., Bjourson A.J., Duddy W., Duguez S.M.R. (2019). A systematic review of suggested molecular strata, biomarkers and their tissue sources in ALS.. Front. Neurol..

[248] Foggin S., Mesquita-Ribeiro R., Dajas-Bailador F., Layfield R. (2019). Biological significance of microRNA biomarkers in ALS-innocent bystanders or disease culprits?. Front. Neurol..

[249] Ravnik-Glavač M., Glavač D. (2020). Circulating RNAs as potential Biomarkers in amyotrophic lateral sclerosis.. Int. J. Mol. Sci..

[250] Angelova M.T., Dimitrova D.G., Dinges N., Lence T., Worpenberg L., Carré C., Roignant J.Y. (2018). The emerging field of epitranscriptomics in neurodevelopmental and neuronal disorders.. Front. Bioeng. Biotechnol..

[251] Hosaka T., Tsuji H., Tamaoka A. (2021). Biomolecular modifications linked to oxidative stress in amyotrophic lateral sclerosis: determining promising biomarkers related to oxidative stress.. Processes (Basel).

[252] Hideyama T., Yamashita T., Aizawa H., Tsuji S., Kakita A., Takahashi H., Kwak S. (2012). Profound downregulation of the RNA editing enzyme ADAR2 in ALS spinal motor neurons.. Neurobiol. Dis..

[253] Sasaki S., Yamashita T., Shin K. (2015). Autophagy in spinal motor neurons of conditional ADAR2-knockout mice: An implication for a role of calcium in increased autophagy flux in ALS.. Neurosci. Lett..

[254] Moore S., Alsop E., Lorenzini I., Starr A., Rabichow B.E., Mendez E., Levy J.L., Burciu C., Reiman R., Chew J., Belzil V.V., W. Dickson D. (2019). Robertson, J.; Staats, K.A.; Ichida, J.K.; Petrucelli, L.; Van Keuren-Jensen, K.; Sattler, R. ADAR2 mislocalization and widespread RNA editing aberrations in C9orf72-mediated ALS/FTD.. Acta Neuropathol..

[255] Quoibion A. (2017). m6A RNA Methylation and TARDBP, a Gene Implicated in Amyotrophic Lateral Sclerosis. McGill University: Montréal.

[256] Kim H.J., Kim N.C., Wang Y.D., Scarborough E.A., Moore J., Diaz Z., MacLea K.S., Freibaum B., Li S., Molliex A., Kanagaraj A.P., Carter R., Boylan K.B., Wojtas A.M., Rademakers R., Pinkus J.L., Greenberg S.A., Trojanowski J.Q., Traynor B.J., Smith B.N., Topp S., Gkazi A.S., Miller J., Shaw C.E., Kottlors M., Kirschner J., Pestronk A., Li Y.R., Ford A.F., Gitler A.D., Benatar M., King O.D., Kimonis V.E., Ross E.D., Weihl C.C., Shorter J., Taylor J.P. (2013). Mutations in prion-like domains in hnRNPA2B1 and hnRNPA1 cause multisystem proteinopathy and ALS.. Nature.

[257] Mitropoulos K., Merkouri P.E., Xiromerisiou G., Balasopoulou A., Charalampidou K., Galani V., Zafeiri K.V., Dardiotis E., Ralli S., Deretzi G., John A., Kydonopoulou K., Papadopoulou E., di Pardo A., Akcimen F., Loizedda A., Dobričić V., Novaković I., Kostić V.S., Mizzi C., Peters B.A., Basak N., Orrù S., Kiskinis E., Cooper D.N., Gerou S., Drmanac R., Bartsakoulia M., Tsermpini E.E., Hadjigeorgiou G.M., Ali B.R., Katsila T., Patrinos G.P. (2017). Genomic variants in the FTO gene are associated with sporadic amyotrophic lateral sclerosis in Greek patients.. Hum. Genomics.

[258] Blanco S., Dietmann S., Flores J.V., Hussain S., Kutter C., Humphreys P., Lukk M., Lombard P., Treps L., Popis M., Kellner S., Hölter S.M., Garrett L., Wurst W., Becker L., Klopstock T., Fuchs H., Gailus-Durner V., Hrabĕ de Angelis M., Káradóttir R.T., Helm M., Ule J., Gleeson J.G., Odom D.T., Frye M. (2014). Aberrant methylation of t RNA s links cellular stress to neuro‐developmental disorders.. EMBO J..

[259] Hartung T., Rhein M., Kalmbach N., Thau-Habermann N., Naujock M., Müschen L., Frieling H., Sterneckert J., Hermann A., Wegner F., Petri S. (2021). Methylation and expression of mutant FUS in motor neurons differentiated from induced pluripotent stem cells from ALS patients.. Front. Cell Dev. Biol..

[260] Hogg M.C., Rayner M., Susdalzew S., Monsefi N., Crivello M., Woods I., Resler A., Blackbourn L., Fabbrizio P., Trolese M.C., Nardo G., Bendotti C., van den Berg L.H., van Es M.A., Prehn J.H.M. (2020). 5′ValCAC tRNA fragment generated as part of a protective angiogenin response provides prognostic value in amyotrophic lateral sclerosis.. Brain Commun..

[261] Taylor R., Hamid F., Fielding T., Gordon P.M., Maloney M., Makeyev E.V., Houart C. (2022). Prematurely terminated intron-retaining mRNAs invade axons in SFPQ null-driven neurodegeneration and are a hallmark of ALS.. Nat. Commun..

[262] Mead R.J., Shan N., Reiser H.J., Marshall F., Shaw P.J. (2023). Amyotrophic lateral sclerosis: a neurodegenerative disorder poised for successful therapeutic translation.. Nat. Rev. Drug Discov..

[263] Corcia P., Beltran S., Bakkouche S.E., Couratier P. (2021). Therapeutic news in ALS.. Rev. Neurol..

[264] Ketabforoush A.H.M.E., Chegini R., Barati S., Tahmasebi F., Moghisseh B., Joghataei M.T., Faghihi F., Azedi F. (2023). Masitinib: The promising actor in the next season of the amyotrophic lateral sclerosis treatment series.. Biomed. Pharmacother..

[265] Eisen A., Kim S., Pant B. (1992). Amyotrophic lateral sclerosis (ALS): A phylogenetic disease of the corticomotoneuron?. Muscle Nerve.

[266] Marques C., Burg T., Scekic-Zahirovic J., Fischer M., Rouaux C. (2021). Upper and lower motor neuron degenerations are somatotopically related and temporally ordered in the Sod1 mouse model of amyotrophic lateral sclerosis.. Brain Sci..

[267] Lu S., Hu J., Arogundade O.A., Goginashvili A., Vazquez-Sanchez S., Diedrich J.K., Gu J., Blum J., Oung S., Ye Q., Yu H., Ravits J., Liu C., Yates J.R., Cleveland D.W. (2022). Heat-shock chaperone HSPB1 regulates cytoplasmic TDP-43 phase separation and liquid-to-gel transition.. Nat. Cell Biol..

[268] Pradhan J., Noakes P.G., Bellingham M.C. (2019). The role of altered BDNF/TrkB signaling in amyotrophic lateral sclerosis.. Front. Cell. Neurosci..

[269] Paganoni S., Berry J.D., Quintana M., Macklin E., Saville B.R., Detry M.A., Chase M., Sherman A.V., Yu H., Drake K., Andrews J., Shefner J., Chibnik L.B., Vestrucci M., Cudkowicz M.E. (2022). Adaptive platform trials to transform amyotrophic lateral sclerosis therapy development.. Ann. Neurol..

[270] Jacquez G.M., Sabel C.E., Shi C. (2015). Genetic GIScience: toward a place-based synthesis of the genome, exposome, and behavome.. Ann. Assoc. Am. Geogr..

[271] Fidler J.A., Treleaven C.M., Frakes A., Tamsett T.J., McCrate M., Cheng S.H., Shihabuddin L.S., Kaspar B.K., Dodge J.C. (2011). Disease progression in a mouse model of amyotrophic lateral sclerosis: the influence of chronic stress and corticosterone.. FASEB J..

[272] Calabrese V., Mancuso C., Calvani M., Rizzarelli E., Butterfield D.A., Giuffrida Stella A.M. (2007). Nitric oxide in the central nervous system: neuroprotection versus neurotoxicity.. Nat. Rev. Neurosci..

[273] Calabrese V., Cornelius C., Dinkova-Kostova A.T., Calabrese E.J., Mattson M.P. (2010). Cellular stress responses, the hormesis paradigm, and vitagenes: novel targets for therapeutic intervention in neurodegenerative disorders.. Antioxid. Redox Signal..

[274] Calabrese V., Cornelius C., Dinkova-Kostova A.T., Calabrese E.J. (2009). Vitagenes, cellular stress response, and acetylcarnitine: Relevance to hormesis.. Biofactors.

[275] Anzilotti S., Brancaccio P., Simeone G., Valsecchi V., Vinciguerra A., Secondo A., Petrozziello T., Guida N., Sirabella R., Cuomo O., Cepparulo P., Herchuelz A., Amoroso S., Di Renzo G., Annunziato L., Pignataro G. (2018). Preconditioning, induced by sub-toxic dose of the neurotoxin L-BMAA, delays ALS progression in mice and prevents Na^+^/Ca^2+^ exchanger 3 downregulation.. Cell Death Dis..

[276] Siracusa R., Scuto M., Fusco R., Trovato A., Ontario M.L., Crea R., Di Paola R., Cuzzocrea S., Calabrese V. (2020). Anti-inflammatory and anti-oxidant activity of Hidrox® in rotenone-induced Parkinson’s disease in mice.. Antioxidants.

[277] Kim D., Nguyen M.D., Dobbin M.M., Fischer A., Sananbenesi F., Rodgers J.T., Delalle I., Baur J.A., Sui G., Armour S.M., Puigserver P., Sinclair D.A., Tsai L.H. (2007). SIRT1 deacetylase protects against neurodegeneration in models for Alzheimer’s disease and amyotrophic lateral sclerosis.. EMBO J..

[278] Calabrese E.J., Calabrese V., Giordano J. (2022). Brain health promotion: Tactics within a strategic approach based upon valid, yet evolving scientific evidence.. Mech. Ageing Dev..

[279] Bauer P.O. (2016). Methylation of C9orf72 expansion reduces RNA foci formation and dipeptide-repeat proteins expression in cells.. Neurosci. Lett..

[280] Lam F., Chu J., Choi J.S., Cao C., Hitchens T.K., Silverman S.K. (2021). Epigenetic MRI: noninvasive imaging of DNA methylation in the brain.. BioRxiv.

[281] Choi S.Y., Lee J.H., Chung A.Y., Jo Y., Shin J., Park H.C., Kim H., Lopez-Gonzalez R., Ryu J.R., Sun W. (2020). Prevention of mitochondrial impairment by inhibition of protein phosphatase 1 activity in amyotrophic lateral sclerosis.. Cell Death Dis..

[282] Paganoni S., Hendrix S., Dickson S.P., Knowlton N., Berry J.D., Elliott M.A. (2022). Effect of sodium phenylbutyrate/taurursodiol on tracheostomy/ventilation-free survival and hospitalisation in amyotrophic lateral sclerosis: Long-term results from the Centaur trial. J Neurol.. Neurosurg. Amp. Psychiatry.

[283] Klingl Y.E., Pakravan D., Van Den B.L. (2020). Opportunities for histone deacetylase inhibition in amyotrophic lateral sclerosis.. Br. J. Pharmacol..

[284] Xia Z., Tang M., Ma J., Zhang H., Gimple R.C., Prager B.C., Tang H., Sun C., Liu F., Lin P., Mei Y., Du R., Rich J.N., Xie Q. (2021). Epitranscriptomic editing of the RNA N6-methyladenosine modification by dCasRx conjugated methyltransferase and demethylase.. Nucleic Acids Res..

[285] Batra R., Nelles D.A., Pirie E., Blue S.M., Marina R.J., Wang H., Chaim I.A., Thomas J.D., Zhang N., Nguyen V., Aigner S., Markmiller S., Xia G., Corbett K.D., Swanson M.S., Yeo G.W. (2017). Elimination of toxic microsatellite repeat expansion RNA by RNA-targeting Cas9.. Cell.

[286] Keogh M.J., Wei W., Aryaman J., Wilson I., Talbot K., Turner M.R., McKenzie C.A., Troakes C., Attems J., Smith C., Al Sarraj S., Morris C.M., Ansorge O., Pickering-Brown S., Jones N., Ironside J.W., Chinnery P.F. (2018). Oligogenic genetic variation of neurodegenerative disease genes in 980 postmortem human brains.. J. Neurol. Neurosurg. Psychiatry.

[287] Chiò A., Mazzini L., D’Alfonso S., Corrado L., Canosa A., Moglia C., Manera U., Bersano E., Brunetti M., Barberis M., Veldink J.H., van den Berg L.H., Pearce N., Sproviero W., McLaughlin R., Vajda A., Hardiman O., Rooney J., Mora G., Calvo A., Al-Chalabi A. (2018). The multistep hypothesis of ALS revisited.. Neurology.

